# Exome Sequencing Reveals the Genetic Architecture of Non‐syndromic Orofacial Clefts and Identifies *BOC* as a Novel Causal Gene

**DOI:** 10.1002/advs.202412073

**Published:** 2025-06-04

**Authors:** Qing He, Min Yu, Yuhua Jiao, Yizhu Xu, Xuqin Liang, Wenbin Huang, Linping Xu, Yuxia Hou, Zhanping Ren, Beile Lyu, Zhenwei Qian, Pengpeng Liu, Jing Zhou, Huimei Huang, Chunyan Yin, Huaxiang Zhao, Yi Ding

**Affiliations:** ^1^ Department of Physiology and Pathophysiology School of Basic Medical Sciences Health Science Center Xi'an Jiaotong University Xi'an Shaanxi 710061 China; ^2^ Key Laboratory of Shaanxi Province for Craniofacial Precision Medicine Research College of Stomatology Xi'an Jiaotong University Xi'an Shaanxi 710004 China; ^3^ Department of Prosthodontics Peking University School and Hospital of Stomatology National Center for Stomatology National Clinical Research Center for Oral Diseases National Engineering Research Center of Oral Biomaterials and Digital Medical Devices Beijing 100081 China; ^4^ Department of Orthodontics College of Stomatology Xi'an Jiaotong University Xi'an Shaanxi 710004 China; ^5^ Department of Orthodontics Stomatological Center Peking University Shenzhen Hospital Shenzhen Guangdong 518055 China; ^6^ Department of Cleft Palate‐Craniofacial Surgery College of Stomatology Xi'an Jiaotong University Xi'an Shaanxi 710004 China; ^7^ State Key Laboratory of Cellular Stress Biology School of Life Sciences Xiamen University Xiamen 361100 China; ^8^ Peking University 302 Clinical Medical School Beijing 100039 China; ^9^ Institute of Advanced Biotechnology Institute of Homeostatic Medicine and School of Medicine Southern University of Science and Technology Shenzhen 518055 China; ^10^ Department of Pediatrics The Second Affiliated Hospital of Xi'an Jiaotong University Xi'an Shaanxi 710004 China; ^11^ Department of Nephrology Xi'an Children's Hospital The Affiliated Children's Hospital Xi'an Jiaotong University Xi'an Shaanxi 710003 China

**Keywords:** *BOC*, epistatic antagonism, genetic architecture, non‐syndromic orofacial clefts, SHH signaling

## Abstract

Nonsyndromic orofacial clefts (NSOFCs) are the most common human craniofacial defects. Genetic factors play a critical role in the pathogenesis of NSOFCs. However, known causal genes only explain a minority of the estimated heritability. To unveil the underlying genetic architecture, exome sequencing is performed on 214 sporadic patients with NSOFCs. The findings substantiate the genetic and allelic heterogeneity of NSOFCs and underscore the crucial role of dysregulation of OFC‐related signaling pathways in the occurrence of NSOFCs. Besides, the candidate variants discovered provide a fruitful resource for further genetic studies. Particularly, three *BOC* missense variants (p.R407W, p.G436S, and p.D1018N) are identified in three unrelated cases with cleft palate. In parallel, a *BOC* nonsense variant (p.R681X), co‐segregating with a *GLI2* missense variant (p.A543G), is identified in a multiplex family with microform cleft lip. Functional studies demonstrate while the four *BOC* variants are hypomorphic alleles, the *GLI2* variant is a hypermorphic allele. The counteraction between *BOC* p.R681X allele and *GLI2* p.A543G allele accounts for the mild phenotype in the multiplex family. Thus, this study establishes *BOC* as a novel causal gene and implicates a two‐locus model of inheritance via the epistatic antagonism of two SHH pathway variants in NSOFCs.

## Graphical abstract

Nonsyndromic orofacial clefts (NSOFCs) are the most common craniofacial defects. Exome sequencing of 214 sporadic cases sheds new light on its genetic architecture and identifies many candidate pathogenic variants. Furthermore, functional studies establish *BOC* as a novel causal gene and reveal an unusual two‐locus model of inheritance via the epistatic antagonism of two SHH pathway variants in NSOFCs.

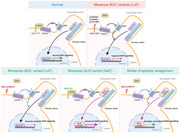

## 1 Introduction

Nonsyndromic orofacial clefts (NSOFCs), including cleft lip (CL), cleft lip and palate (CLP), and cleft palate (CP), are among the most common human congenital structural birth defects, affecting on average 1/800 live births worldwide.^[^ ^1^ ^]^ In addition to the dysfunction of the lips and oral cavity, individuals with NSOFCs also have significant impairment of speech, hearing, appearance, and cognition, which cause high morbidity and mortality throughout life. To restore optimal function, children born with NSOFCs need to undergo multiple medical procedures from birth until adulthood, including maxillofacial surgeries, orthodontic treatment, feeding therapy, language therapy, and psychotherapy. Therefore, NSOFCs constitute substantial healthcare and financial burdens on the affected individuals, their families, and society.^[^ ^1a^ ^]^

The mammalian face is initially made up of five prominences: a central frontonasal prominence, two paired maxillary prominences, and two paired mandibular prominences.^[^ ^2^ ^]^ The development of the upper lip and primary palate commences upon the division of the frontonasal prominence into paired lateral and medial nasal processes by the formation of the nasal pits. Subsequently, the fusion of the medial nasal, lateral nasal, and maxillary processes creates the upper lip, and the fusion of paired medial nasal processes with each other at the midline gives rise to the philtrum and the primary palate. Then the secondary palate forms from palatal shelves derived from the maxillary prominences, which go through a series of morphogenetic movements that include vertical growth, elevation, horizontal growth, adhesion, and final fusion.^[^ ^3^ ^]^ These developmental events are closely coordinated by signals from a group of conserved growth factors that include SHH, FGF, WNT, and TGF‐β/BMP.^[^ ^4^ ^]^ Perturbation of the signaling pathways initiated by these signals can disrupt lip and palate development and cause orofacial clefts in both animal models and humans.^[^ ^4^ ^]^

Genetic and epidemiological studies have clarified that NSOFCs have a multifactorial etiology, involving genetic and environmental factors and the interplay between them.^[^ ^2b^ ^]^ Among them, genetic factors, such as risk alleles and their interactions, indubitably play a prominent role.^[^ ^2b^ ^]^ Several genetic approaches, including linkage, candidate gene association studies, genome‐wide association studies (GWAS), exome sequencing (ES), and whole‐genome sequencing (WGS), have been applied to dissect the genetic bases of NSOFCs.^[^ ^5^ ^]^ While GWAS have successfully identified dozens of common variant loci (minor allele frequency > 1%), these collectively account for only 10–25% of NSOFC heritability.^[^ ^5^, ^6^ ^]^ Importantly, many of these GWAS signals are located in non‐coding regulatory regions, making it challenging to pinpoint the specific causal genes and elucidate the underlying pathogenic mechanisms.^[^ ^7^ ^]^ Compelling evidence now demonstrates that rare variants (minor allele frequency <0.1%) significantly contribute to NSOFC pathogenesis.^[^ ^8^ ^]^ Yet, a large portion of disease‐linked rare variants cluster within known OFC genes and they can explain only a fraction of the missing heritability.^[^ ^9^ ^]^ The remaining heritability likely stems from pathogenic rare variants in novel causal genes, complex genetic interactions between risk alleles, and gene‐environment interactions that modulate disease susceptibility.

To further explore the genetic architecture of NSOFCs, we analyzed 214 sporadic patients with NSOFCs by ES. Our findings confirmed the genetic and allelic heterogeneity of NSOFCs and underscored the significance of OFC‐related signaling pathway variants in NSOFCs. More importantly, we uncovered many novel variants that provide a fruitful resource for future research. Among these novel variants, three rare monoallelic missense variants in *BOC* occurring in patients with CP were functionally validated to be loss‐of‐function mutations. Besides, our ES analysis of a multiplex family with microform cleft lip identified a monoallelic hypomorphic *BOC* nonsense variant, which was antagonized epistatically by a co‐transmitting monoallelic hypermorphic *GLI2* missense variant in the carriers. This genetic counteraction accounts for the mild phenotype in the multiplex family. These results identify *BOC* as a novel causal gene and reveal, for the first time to our knowledge, a two‐locus mode of inheritance via epistatic antagonism between two variants in one OFC‐related signaling pathway in NSOFCs.

## 2 Results

### 2.1 Exome Sequencing Analysis of 214 Sporadic Patients With NSOFCs

**Figure 1:**
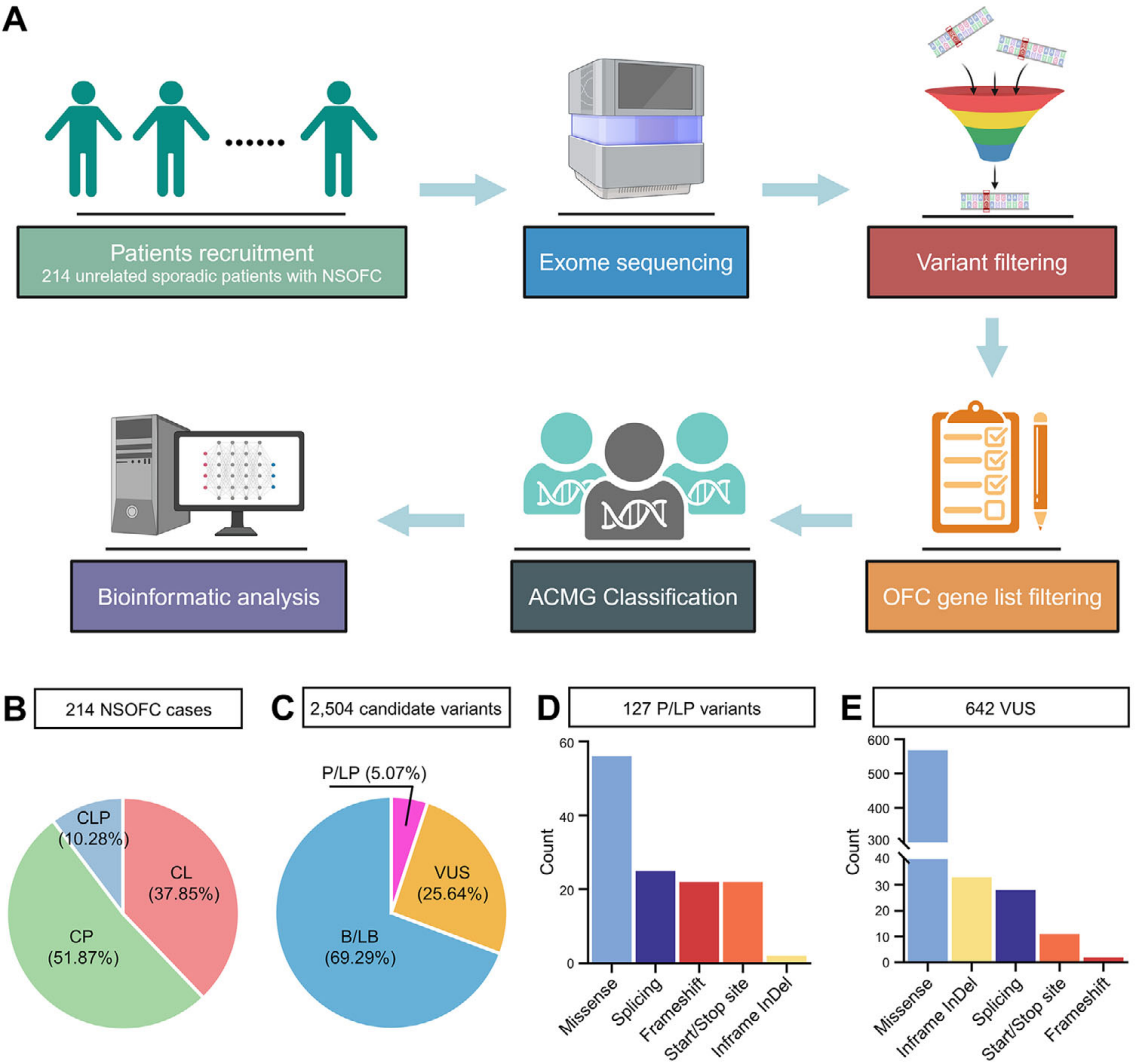
Identification of candidate rare variants in sporadic patients with NSOFCs. (A) Flowchart for screening candidate rare variants in sporadic patients with NSOFCs. (B) The 214 NSOFC patients consist of 81 (37.85%) cases with cleft lip (CL), 111 (51.87%) cases with cleft palate (CP), and 22 (10.28%) cases with cleft lip and palate (CLP). (C) The 2504 candidate rare variants comprise 127 (5.07%) P/LP variants, 642 (25.64%) VUS, and 1735 (69.29%) B/LB variants. (D and E) Proportions of different variant types in P/LP variants (D) and VUS (E).

Given that NSOFCs are typically sporadic in nature and only a small fraction of affected individuals have a family history,^[^ ^10^ ^]^ we speculate that ES interrogation of sporadic cases, similar to familial cases, will also aid in elucidating the genetic architecture of NSOFCs. In total, we recruited 214 sporadic patients, including 81 cases with CL (38 males and 43 females), 111 cases with CP (53 males and 58 females), and 22 cases with CLP (13 males and 9 females), from Western China and performed ES on them (**Figure** **1**A,B, and Table S1).

It was recently demonstrated that a set of 418 OFC genes was productive in prioritizing variants for the genetic diagnosis of patients with OFCs.^[^ ^9^ ^]^ When scrutinizing these 418 OFC genes, we noticed that 8.37% (35/418) of them encode members of OFC‐related SHH, FGF, WNT, and TGF‐β/BMP signaling pathways, suggesting a critical role of dysregulation of these pathways in the pathogenesis of OFCs. Therefore, we reasoned that a better diagnostic yield would be achieved if this set of 418 OFC genes for variant prioritization could be expanded to include a more comprehensive list of 154 genes encoding all key members of OFC‐related signaling pathways (Table S2). This approach resulted in a new list of 537 unique genes after the removal of 35 duplicated genes, which was used to screen for candidate variants in our cohort.

After variants filtering (Figures S1, S2), we identified a total of 2504 candidate variants in 457 genes comprised of 367 genes from the 418 OFC genes and 122 genes from the 154 OFC‐related signaling pathway genes (Tables S2, S3). The 2504 variants consisted of 127 (5.07%) pathogenic/likely pathogenic (P/LP) variants in 80 genes from 89 cases, 642 (25.64%) variants of uncertain significance (VUS) in 302 genes from 205 cases, and 1735 (69.29%) benign/likely benign (B/LB) variants in 392 genes from 214 cases (Figure 1C and Table S3). These 2504 variants could be categorized into five types: missense, splicing, inframe indel, start/stop site, and frameshift. The 127 P/LP variants were dominated by those presumed to be loss‐of‐function variants: 22 frameshift variants, 22 start/stop site variants, 25 splicing variants, 56 missense variants, and 2 inframe insertion/deletion (indel) variants (Figure 1D). There were over 5 times more VUS than P/LP variants, the majority of which were missense variants, reflecting the difficulty in interpreting missense variants without functional evidence (Figure 1E). Some frameshift variants, start/stop site variants, and inframe indel variants were also classified as VUS, but they only constitute a relatively small fraction (Figure 1E).

Although not interpreted as LP/P variants in the American College of Medical Genetics and Genomics (ACMG) framework, VUS can be truly disease‐causing.^[^ ^9^ ^]^ Therefore, we included VUS in our further analysis. To gain further insights into the pool of P/LP and VUS variants, we performed Gene Ontology (GO) and Kyoto Encyclopedia of Genes and Genomes (KEGG) pathway enrichment analysis.^[^ ^11^ ^]^ The most enriched biological processes (BP) in GO terms centered on embryonic development and organogenesis (Figure S3A). Additionally, the most relevant cellular component (CC) in GO terms involves cilia structure and function (Figure S3B). In the analysis of GO‐molecular function (MF) and KEGG pathways, the Hippo, WNT, MTOR, TGF‐β, and HH signaling pathways were the most enriched, along with terms related to the molecular functions of membrane receptor proteins (Figure S3C,D). Interestingly, all five signaling pathways and cilia are known to regulate each other during embryonic development and organogenesis, including lip and palate development.

### 2.2 Genetic and Allelic Heterogeneity of NSOFCs

**Figure 2:**
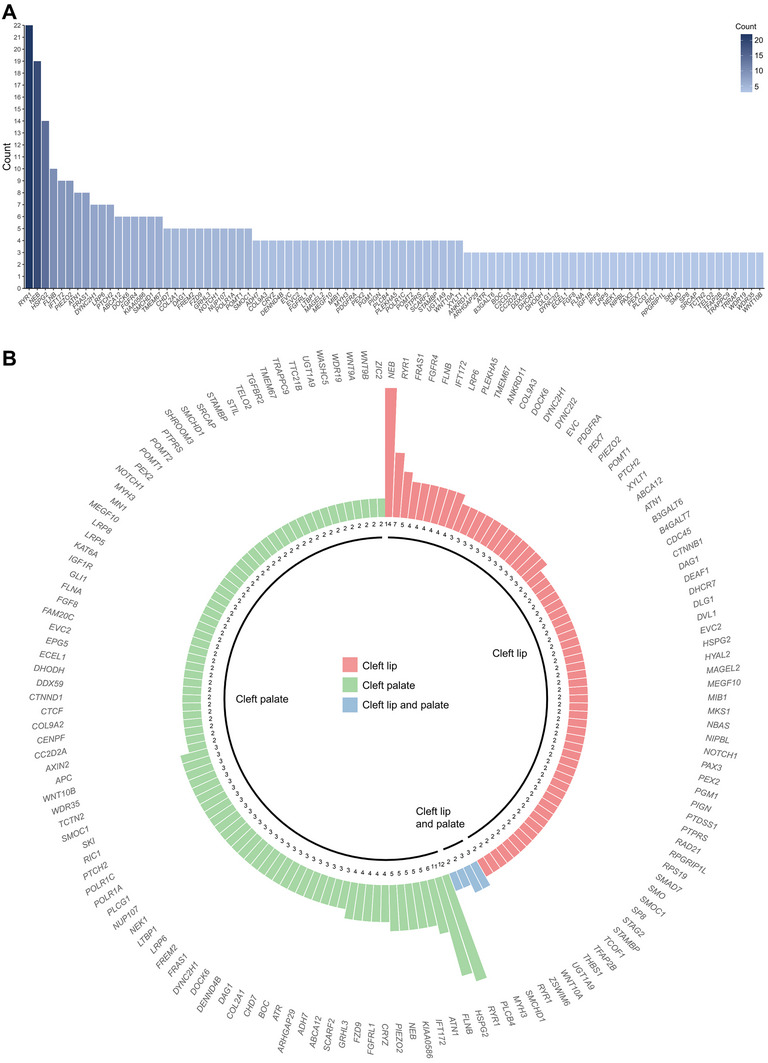
NSOFCs display genetic and allelic heterogeneity. (A) 91 genes with P/LP variants and VUS are detected in at least three cases. The *y*‐axis indicates the number of cases. (B) 151 genes were highlighted due to their recurrent mutation in at least two independent cases, selected from the total 324 genes harboring P/LP variants and VUS. Genes are categorized by cleft subtype: pink for cleft lip, green for cleft palate, and blue for cleft lip and palate. Each bar represents a gene and the height of the bar represents the number of cases carrying variants of the gene, as denoted by numbers below the bar. Refer to Table S 3 for detailed information.

Of the 324 genes harboring P/LP variants and VUS, 91 (28%) genes were recurrently mutated, with three or more different variants detected in at least three unrelated cases. *RYR1* was the most frequently mutated gene, with 21 different variants identified in 22 cases, followed by *NEB*, *HSPG2*, and *FLNB*. Deleterious variants of some of these recurrently mutated genes, such as *FLNB* and *ARHGAP29*, have already been demonstrated to be causal for OFCs.^[^ ^8^, ^12^ ^]^ In contrast, variants in other genes, like *BOC* (with three different variants identified in three cases) and *FGFR4* (with five distinct variants identified in six cases), have not yet been associated with the occurrence of NSOFCs and thus may represent novel risk alleles (**Figure** **2**A). The identification of a high proportion of candidate genes with multiple variants in our cohort underscores the allelic heterogeneity of NSOFCs.

Previous studies have suggested differences in the genetic architecture of specific OFC subtypes.^[^ ^13^ ^]^ Therefore, we stratified our case cohort to test for differences in genes harboring P/LP variants or VUS across CL, CP, and CLP subtypes. Among the 324 genes with P/LP variants or VUS, variants in 187, 229, and 62 genes were identified in CL, CP, and CLP cases, respectively (Figure S4A). Variants in 104 genes were shared between CL and CP cases, 42 genes between CP and CLP, and 33 genes between CL and CLP (Figure S4A). 25 genes harbored variants shared across all three OFC subtypes, but only two contained P/LP variants (Figure S4A,B). As explicitly delineated in Figure 2B, while variants in *RYR1* were identified in cases from all three OFC subtypes (seven CL cases, 12 CP cases, and three CLP cases) and variants in *FLNB* were identified from both CL and CP cases (four CL case and six CP case), variants in *BOC* occurred exclusively in CP cases (three CP cases) (Figure 2B). These observations support the view that both genetic heterogeneity and overlap exist across the three OFC subtypes.^[^ ^13^ ^]^

### 2.3 Enrichment of OFC‐Related Signaling Pathway Variants

**Figure 3:**
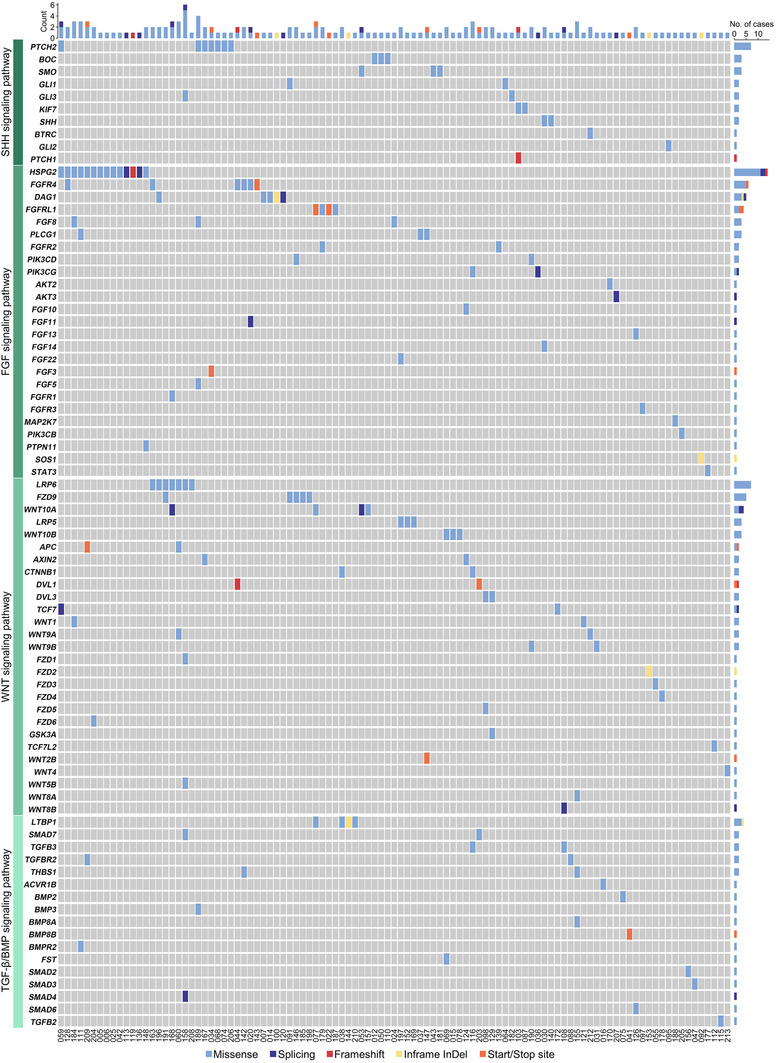
High enrichment of SHH, FGF, WNT, and TGF‐β/BMP signaling pathway variants. Each vertical bar represents a patient (case identifiers are shown at the bottom) and is colored according to variant types and corresponding mutated genes harbored by the patient. The variant types are indicated by the colors shown at the bottom of the graph. The bars on the top indicate the numbers and types of variants harbored by the patient. The bars on the right indicate the number of patients that harbor variants in the genes shown on the left. Refer to Table S4 for detailed information.

We observed that 48.13% (103/214) of the patients in our cohort carried at least one variant in genes encoding components of OFC‐related SHH, FGF, WNT, and TGF‐β/BMP pathways (**Figure** **3** and Table S4). Notably, there were 11 patients that harbored at least three variants in three or more different genes. As seen in Case‐077 with CP, this patient carried three variants in *FGFRL1* (c.1506T>G, p.Y502X), *WNT10A* (c.499G>C, p.E167N), and *LTBP1* (c.607C>T, p.R203W) that encode members of FGF, WNT, and TGF‐β pathways. Additionally, Case‐060 with CP also carried three variants in *WNT9A* (c.682A>T, p.T228S), *LRP6* (c.3887C>T, p.A1296V), and *APC* (c.3232T>G, p.Y1078D) that all encode WNT pathway members. Meanwhile, there were 29 patients who harbored two variants in two different genes. As an illustration, Case‐034 with CL carried two variants in *PTCH2* (c.1670G>A, p.R557H) and *FGF3* (c.310C>T, p.R104X) that encode a receptor and a ligand of the SHH and FGF pathways, respectively. Another example was Case‐103 with CP that carried two variants in *PTCH1* (c.2332dupA, p.T778fs) and *KIF7* (c.250G>A, p.E84K) that both act in the SHH signaling pathway (Figure 3 and Table S4).

These results indicate that genetic variations‐mediated perturbation of the OFC‐related signaling pathways and their crosstalk may contribute significantly to the occurrence of NSOFCs. Besides, genetic interactions between risk alleles in the same pathway and their potential synergism or antagonism may play an unanticipated role.

### 2.4 Characterization of *BOC* Variants In Sporadic and Familial Cases with NSOFCs

**Figure 4:**
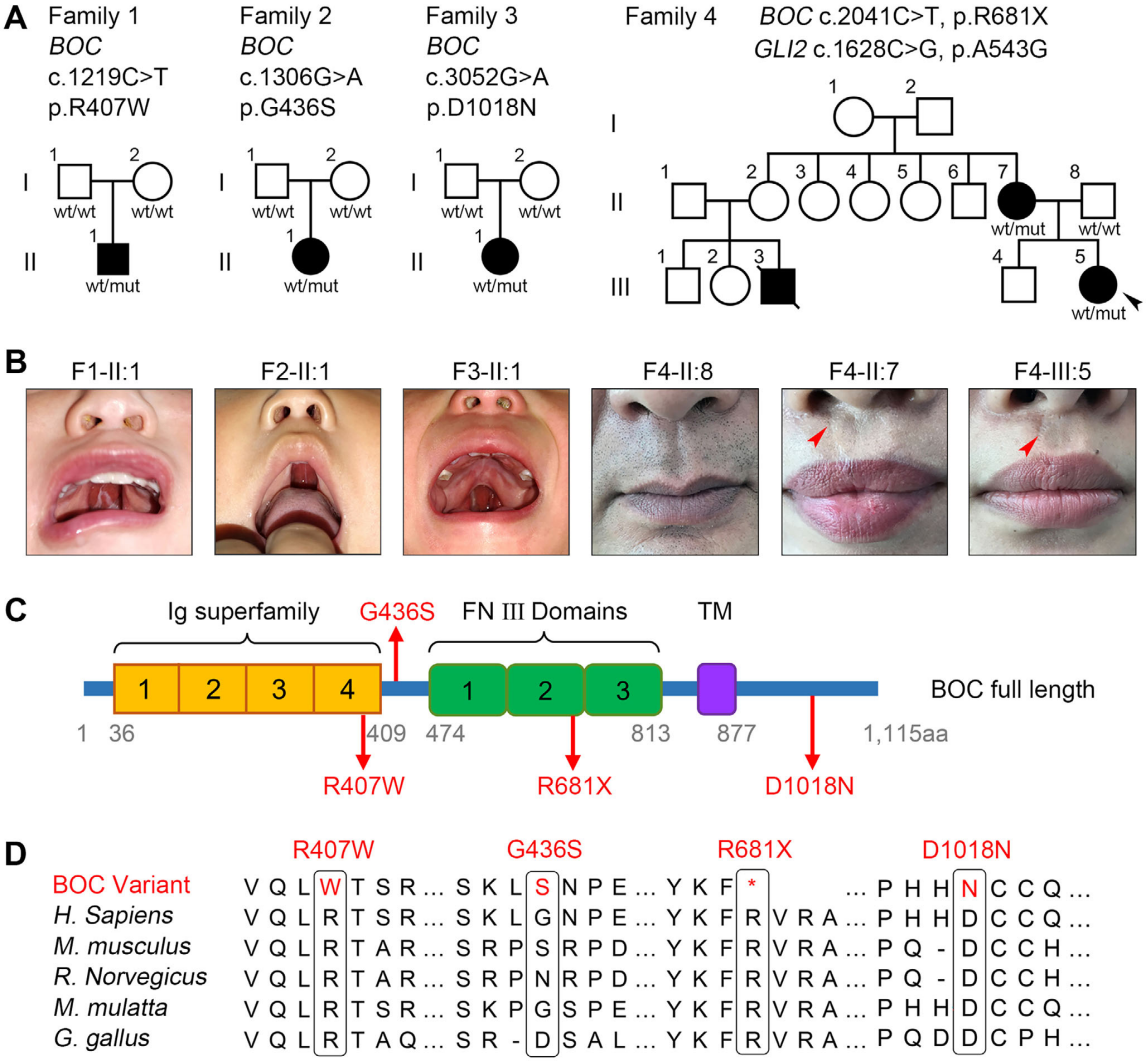
Identification of rare heterozygous variants in *BOC* in three parent–offspring trios and one multiplex family with NSOFCs. (A) Pedigrees of four kindreds with rare *BOC* variants. Squares indicate male family members, circles female family members, open symbols unaffected, black symbols affected, and diagonal slash deceased. While three *de novo* heterozygous *BOC* missense variants (c.1219C>T, p.R407W; c.1306G>A, p.G436S; c.3052G>A, p.D1018N) were identified in Families 1–3, a heterozygous *BOC* nonsense variant (c.2041C>T, p.R681X) cosegregated with a heterozygous *GLI2* missense variant (c.1628C>G, p.A543G) among affected members in Family 4. (B) Images of patients and their family members. Clinically, all probands in Families 1–3 present with cleft palate, while their parents are unaffected. In family 4, both the proband (F4‐III:5) and her mother (F4‐II:7), but not her father (F4‐II:8), present with microform cleft lip (red arrowhead). (C) Schematic of the human *BOC* protein structure showing the Ig superfamily domain, FNIII domains, transmembrane (TM) domain, and the intracellular region. The locations of amino acids affected by p.R407W, p.G436S, p.R681X, and p.D1018N variants are indicated. (D) Multiple sequence alignment showing conservation of Arg407, Arg681, and Asp1018 across vertebrate species. Gly436 is not evolutionally conserved.

We noticed a high enrichment of SHH signaling pathway variants in our cohort. Particularly, the *BOC* gene, with three different heterozygous *de novo* missense variants (c.1219C>T, p.R407W; c.1306G>A, p.G436S; c.3052G>A, p.D1018N) carried by three unrelated individuals (F1‐II:1, F2‐II:1, and F3‐II:1) presenting with CP, has not been described in NSOFCs (**Figure** **4**A,B Figure S6A, **Table** **1**, and Tables S3, S5). Meanwhile, in a separate ES study of a multiplex family with NSOFCs, we also identified a previously unrecorded heterozygous nonsense variant in *BOC* (c.2041C>T, p.R681X) co‐segregating with a heterozygous missense variant in *GLI2* (c.1628C>G, p.A543G) in the proband (F4‐III:5) and her affected mother (F4‐II:7). This variant pair followed a dominant inheritance pattern, with both carriers exhibiting microform cleft lip (surgically corrected) and no additional anomalies (Figure 4A and B, Table 1, Figures S5, S6). The proband also had an affected first cousin (F4‐III:3), who exhibited CLP and had died, and no medical autopsy was available (Figure 4A). All the *BOC* variants were rare in the general population (Table 1). While the three missense variants in *BOC* were classified as VUS, the nonsense *BOC* variant was predicted to be a likely pathogenic loss‐of‐function allele (Table 1). These observations prompted us to hypothesize that *BOC* might be a potential causal gene for NSOFCs.

**Table 1:** Genetic and clinical characteristics of patients with *BOC* and *GLI2* variants. NR: Not Reported. NA: Not Applicable. Variant pathogenicity was predicted by SIFT and Polyphen2.

Gene	*BOC* (Family 1‐4) NM_001301861.2				*GLI2* (Family 4) NM_005270.5
Affected individuals	Family 1 II‐1	Family 2 II‐1	Family 3 II‐1	Family 4 II‐7, III‐5	Family 4 II‐7, III‐5
Cleft type	cleft palate	cleft palate	cleft palate	Microform cleft lip	microform cleft lip
cDNA	c.1219C>T	c.1306G>A	c.3052G>A	c.2041C>T	c.1628C>G
Protein	p.R407W	p.G436S	p.D1018N	p.R681X	p.A543G
dbSNP	rs370137874	rs149191848	rs199688330	NR^a)^	rs201279367
East Asian in ALFA	0	0	0	NR	0
Asian in ExAc	0.00102	0.00035	0	NR	0.00116
All GnomAD‐ exomes individuals	0.0002533	0.0003246	0	NR	0.0002347
All GnomAD‐genomes individuals	0.0002868	0.0003189	0	NR	0.0001912
SIFT	deleterious	tolerated	deleterious	NA^b)^	deleterious
PolyPhen‐2	probably damaging	benign	benign	NA	probably damaging

^a)^ NR: Not Reported

^b)^ NA: Not Applicable. Variant pathogenicity was predicted by SIFT and Polyphen2.

We first assessed the pathogenicity of *BOC* variants in silico. Human *BOC* encodes an SHH co‐receptor comprised of an extracellular region of four Immunoglobulin (Ig) repeats followed by a linker region and three Fibronectin type III (FNIII) repeats, a single‐pass transmembrane domain, and an intracellular region.^[^ ^14^ ^]^ Multiple sequence alignment revealed that while Gly436 is not conserved and located in the linker region, both Arg407 and Asp1018 are highly conserved across vertebrate *BOC* orthologues and situated in the fourth Ig superfamily domain and the intracellular domain, respectively (Figure 4C,D). The p.R681X nonsense variant is supposed to produce a secreted protein consisting only of the Ig superfamily domain, the linker region, and the first part of the second FN repeat (Figure 4C,D). Given the critical role of *BOC* in transducing SHH signals, these *BOC* genetic variations might result in aberrant SHH signaling and thus occurrence of NSOFCs.

### 2.5 *BOC* Variants Mimic Loss‐Of‐Function Mutations In Vivo

Zebrafish has offered an ideal model to study the contributions of genetic factors to human orofacial clefts.^[^ ^15^ ^]^ Like in mammals, the zebrafish craniofacial skeleton is largely derived from pharyngeal arches (PAs). After being colonized by cranial neural crest cells, the mandibular arch of PA1 gives rise to Meckel's cartilage and palatoquadrate, and the maxillary arch of PA1 contributes to the ethmoid plate and trabeculae. The Meckel's cartilage acts as the larval lower jaw, while the ethmoid plate (analogous to the mammalian palate) articulates with the retroarticular processes of the palatoquadrate to form the larval upper jaw. PA2 (the hyoid arch) forms the ceratohyal and hyomandibular bones; and PA3‐7 (the brachial arches) give rise to the ceratobranchials, epibranchials, and pharyngobranchials (Figure S7A).^[^ ^15^, ^16^ ^]^

**Figure 5:**
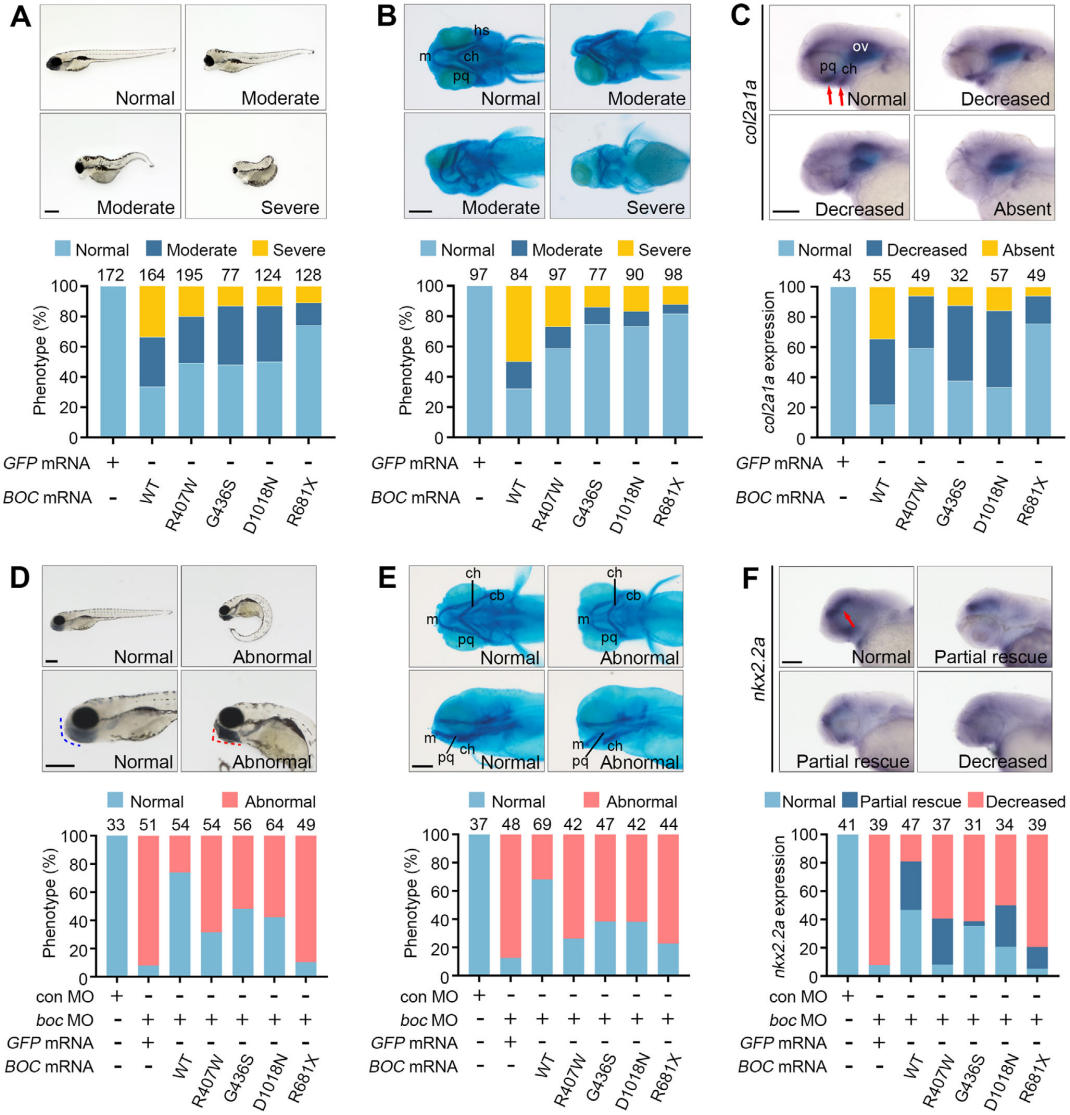
The *BOC* variants behave as loss‐of‐function mutations in zebrafish embryos. (A) *BOC* variants are less active in inducing developmental defects. 1‐cell stage zebrafish embryos were injected with 300 pg of mRNAs encoding WT or p.R407W, p.G436S, p.D1018N or p.R681X *BOC* variants. Phenotypes were quantified at 5 dpf by comparing embryo morphology and counting embryos with the indicated abnormalities across indicated conditions. Normal: no observable defects. Moderate: craniofacial defects, including microcephaly and eye deformities, shortened body length, or severely curved body axis. Severe: spherical or unrecognized main body structure. The number of embryos per condition is indicated on the top of each column. Scale bar, 200 µm. (B) *BOC* variants are less potent to induce craniofacial cartilage defects. Zebrafish embryos were microinjected as in (A) and analyzed by Alcian blue staining. Phenotypes were quantified by comparing embryo morphology and counting embryos with the indicated abnormalities across indicated conditions. Normal: no observable defects. Moderate: morphological and positional abnormalities of Meckel's cartilage, palatoquadrate, and ceratohyal. Severe: absence of Meckel's cartilage, palatoquadrate, or ceratohyal. The number of embryos per condition is indicated on the top of each column. Scale bar, 200 µm. (C) *BOC* variants are less capable of repressing *col2a1a* expression. Zebrafish embryos were microinjected as in (A) and analyzed by WISH for *col2a1a* at 48 hpf. *col2a1a* is normally expressed in the pharyngeal arches and otic vesicles, as indicated by red arrows. *BOC* mainly affects *col2a1a* expression in pharyngeal arches. Ov: otic vesicle. The number of embryos per condition is indicated on the top of each column. Scale bar, 500 um. (D) *BOC* variants are less active in rescuing *boc* MO‐induced developmental defects. 1‐cell stage zebrafish embryos were injected with control MO (8 ng) or *boc* MO (8 ng) together with 150 pg of mRNAs encoding WT or p.R407W, p.G436S, p.D1018N or p.R681X *BOC* variants as indicated. Phenotypes were quantified at 5 dpf by comparing embryo morphology and counting embryos with the indicated abnormalities across indicated conditions. Normal: straight body axes of normal length and properly developed lower jaw, indicated by blue dashed lines. Abnormal: curved body axes and improperly closing the lower jaw, indicated by red dashed lines. The number of embryos per condition is indicated on the top of each column. Scale bar, 200 µm. (E) *BOC* variants are less potent in rescuing *boc* MO‐induced craniofacial cartilage defects. Zebrafish embryos were microinjected as in (D) and analyzed by Alcian blue staining. Phenotypes were quantified by comparing embryo morphology and counting embryos with the indicated abnormalities across indicated conditions. Normal: Meckel's cartilage, palatoquadrate, ceratohyal, and ceratobranchial have normal morphology and relative angles. Abnormal: Meckel's cartilage, palatoquadrate, and ceratohyal deviate ventrally, with the lower jaw in an open configuration and a significantly reduced craniofacial region. The number of embryos per condition is indicated on the top of each column. Scale bar, 200 µm. (F) *BOC* variants are less capable of rescuing *boc* MO‐induced inhibition of *nkx2.2a* expression. Zebrafish embryos were microinjected as in (D) and analyzed by WISH for *nkx2.2a* at 48 hpf. *nkx2.2a* is normally expressed in the forebrain and midbrain, as indicated by the red arrow. The number of embryos per condition is indicated on the top of each column. Scale bar, 500 um.

We first examined the pathogenicity of the *BOC* variants in zebrafish embryos by overexpression. Zebrafish *boc* is expressed in the central nervous system and the pharyngeal arches during embryogenesis, and *boc* mutant fish display axon guidance and forebrain patterning defects and craniofacial cartilage anomalies that include enlarged lower jaws.^[^ ^17^ ^]^ Microinjection of mRNA encoding wild‐type (WT) *BOC* into zebrafish embryos resulted in curved and shortened body axes and a broad spectrum of craniofacial defects, including microcephaly, microphthalmia, and anophthalmia. By comparison, mRNAs encoding p.R407W, p.G436S, p.R681X, and p.D1018N *BOC* variants (hereafter *BOC* variants) were less active to induce these malformations (**Figure** **5**A). We next performed Alcian Blue staining to visualize the craniofacial cartilage. While over 65% of embryos injected with mRNA encoding WT *BOC* exhibited moderate to severe cartilage defects, ranging from deformation to extensive loss, these abnormalities were less pronounced in embryos injected with mRNAs encoding *BOC* variants (Figure 5B). We further tested the effect of *BOC* variants on the expression of *col2a1a*, a chondrocyte marker, during zebrafish embryogenesis. We noticed that over 75% of embryos injected with mRNA encoding WT *BOC* displayed a significant reduction in *col2a1a* expression in the eyes and/or pharyngeal arches. Again, *BOC* variants were less potent in repressing *col2a1a* expression (Figure 5C).

We then utilized a previously described translation‐blocking antisense morpholino oligonucleotides (MO) targeting *boc* to test whether *BOC* variants could effectively rescue the developmental defects induced by *boc* knockdown in zebrafish embryos. As expected, while control MO‐injected embryos exhibited no discernible abnormalities, the *boc* morphants showed curved body axis, enlarged lower jaws, and craniofacial cartilage defects, phenocopying the *boc* mutant zebrafish (Figure S7B,C).^[^ ^17^ ^]^ These *boc* MO‐induced developmental defects, including both axial and craniofacial anomalies, could be largely rescued by co‐injection of mRNA encoding WT *BOC*. In contrast, co‐expression of mRNAs encoding *BOC* variants was much less effective in rescuing these abnormalities (Figure 5D,E). More importantly, *boc* knockdown significantly decreased the expression of *nkx2.2a*, an SHH signaling target highly expressed in the forebrain and midbrain during zebrafish embryogenesis. Co‐injection of mRNAs encoding WT *BOC*, but not *BOC* variants could largely normalize *nkx2.2a* expression (Figure 5F).

These results demonstrate that the *BOC* variants p.R407W, p.G436S, p.R681X, and p.D1018N are bona fide loss‐of‐function mutations in vivo that cannot sustain proper SHH signaling. Among these, p.R681X shows the lowest activity.

### 2.6 *BOC* Variants Alter Protein Membrane Localization and Binding to SHH and PTCH1

**Figure 6:**
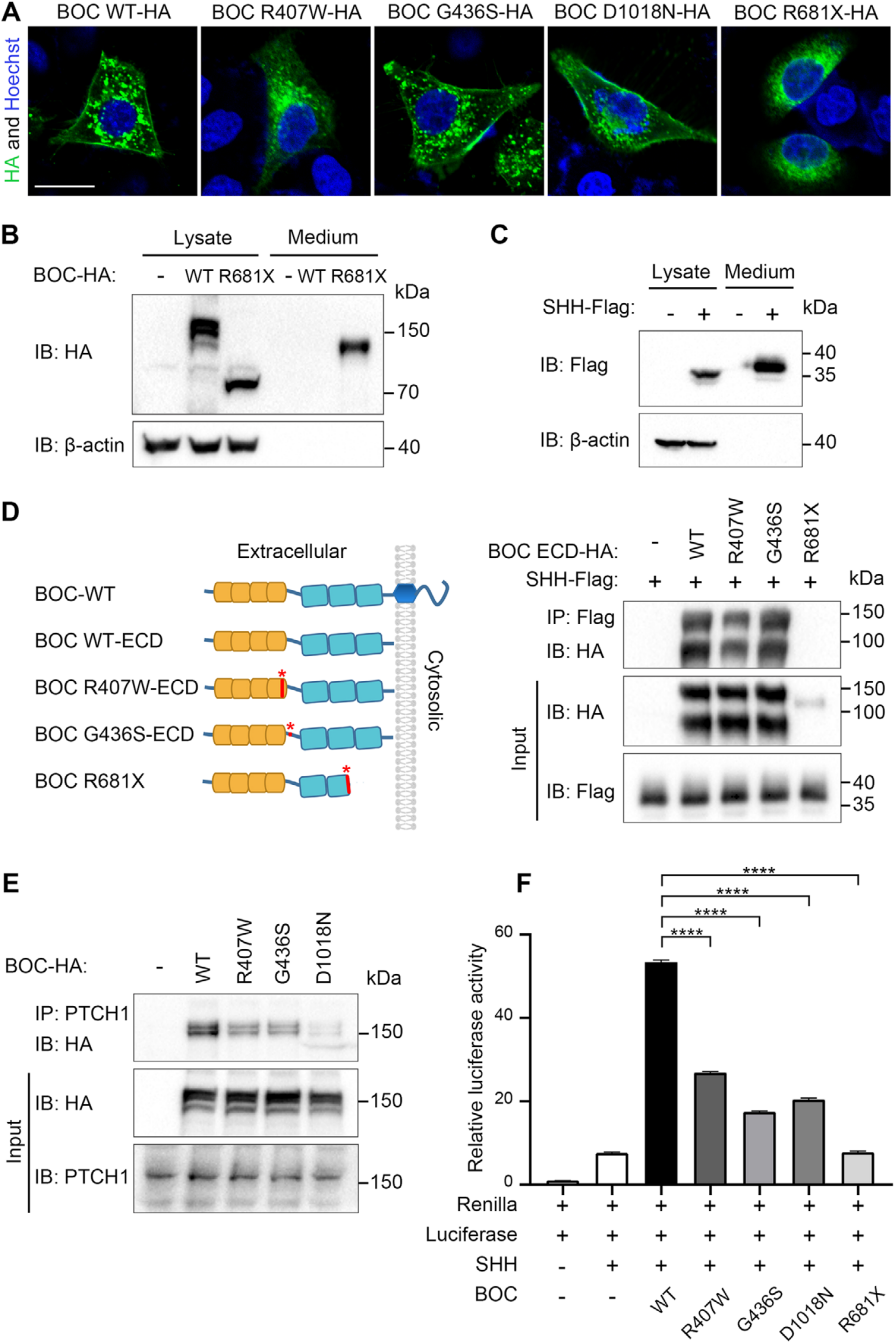
*BOC* variants show defective cell surface localization and impaired binding with SHH and PTCH1. (A) p.R407W and p.R681X variants dislocate *BOC* from the cell surface to the cytoplasm. Hela cells transfected with HA‐tagged WT or mutant *BOC* were processed for immunofluorescence analysis with an HA antibody. p.R407W and p.R681X *BOC* are diffused in the cytoplasm, while the WT *BOC*, as well as p.G436S and p.D1018N *BOC*, is localized on the membrane. Nuclear nucleus was stained with Hoechst. Scale bar, 20um. (B) p.R681X variant results in a secreted protein. HEK293T cells were transfected with WT or mutant *BOC*. 24 h later, cells were serum starved for 20 h. Cell lysate and concentrated medium were analyzed by Western blotting with an HA antibody. β‐actin served as a loading control. (C) Effective secretion of SHH protein. The experiment was performed as in B, except that a Flag‐tagged SHH construct was used. (D) p.R407W and p.R681X variants weaken the binding of *BOC* to SHH. Conditioned media of SHH‐Flag was incubated with conditioned media of extracellular domains (ECD) of WT or mutant *BOC*, followed by a pull‐down assay with a Flag antibody. The WT or mutant *BOC*‐ECD protein bound on the beads was analyzed by Western blotting with an HA antibody. Note that the p.D1018N variant was not analyzed because of the localization of D1018 in the cytoplasmic tail, but not ECD. (E) p.R407W, p.G436S and p.D1018N variants dampen the binding to *BOC* to PTCH1. HEK293T cells were transfected with WT or mutant *BOC* and subjected to immunoprecipitation with a PTCH1 antibody. The WT or mutant *BOC* protein bound on the beads was analyzed by Western blotting with an HA antibody. Note that the p.R681X variant produces a secreted protein and thus its effect on binding to PTCH1 on the cell surface was not analyzed. (F) *BOC* variants display diminished activity in enhancing SHH signaling. NIH3T3 cells were transfected with 8 × 3′Gli‐BS‐delta51‐Luc reporter together with Renilla and WT or mutant *BOC* as indicated. 24 h after transfection, cells were serum starved for 12 h and then stimulated with 100 ng mL^−1^ SHH protein for 16 h. Luciferase and Renilla's activities were measured. Experiments were performed in triplicate. Data are presented as mean ± standard deviation (SD). Statistical significance was assessed by unpaired two‐tailed Student's t‐test. **** *P* < 0.0001.

We sought to determine the molecular and cellular mechanisms underlying the impaired activity of *BOC* variants. Since *BOC* binds both SHH ligand and PTCH1 receptor on the cell surface, we first examined whether these variants affect *BOC* membrane localization. Immunofluorescence assay in Hela cells revealed that p.G436S and p.D1018N *BOC*, similar to WT *BOC*, were primarily localized on the cell membrane (**Figure** **6**A). In contrast, p.R407W and p.R681X *BOC* were predominantly diffused in the cytoplasm, indicating that p.R407W and p.R681X variants disrupted the membrane targeting of *BOC* (Figure 6A). As anticipated, p.R681X *BOC* was copiously secreted to the extracellular milieu upon overexpression in HEK293T cells (Figure 6B), consistent with the loss of membrane localization of this truncating variant. We then examined whether the variants affected the binding of *BOC* with SHH ligand, which was mediated by the third FNIII repeat of *BOC*.^[^ ^14^ ^]^ We generated a carboxyl‐terminal Flag‐tagged human SHH construct and found the encoded SHH protein could be abundantly secreted from transfected HEK293T cells (Figure 6C). We then incubated the conditioned media of SHH protein with conditioned media containing the extracellular domain (ECD) of WT *BOC* or its variants. Pulldown assay revealed that while p.G436S *BOC* showed comparable binding affinity to SHH as the WT *BOC*, p.R407W *BOC* displayed much weaker binding. p.R681X *BOC*, devoid of the third FNIII domain, was expressed and secreted at a much lower level and hardly bound to SHH (Figure 6D). When overexpressed in HEK293T cells, p.R407W, p.G436S, and p.D1018N *BOC* all exhibited decreased association with PTCH1 (Figure 6E). In accordance with these molecular analyses, overexpression of WT *BOC*, but not *BOC* variants, potentiated SHH‐stimulated luciferase activity in NIH3T3 cells (Figure 6F).

To summarize, p.R407W variant dislocates membrane localization of *BOC* and thus impairs its binding to SHH; p.G436S and p.D1018N variants decreased the binding of *BOC* to PTCH1; p.R681X variant abolishes both *BOC* membrane localization and its association with SHH and PTCH1. Therefore, we conclude that these *BOC* variants mimic loss‐of‐function mutations in vitro.

### 2.7 A *GLI2* p.A543G Variant Co‐Segregates and Counteracts With the *BOC* p.R681X Variant in the Multiplex Family

**Figure 7:**
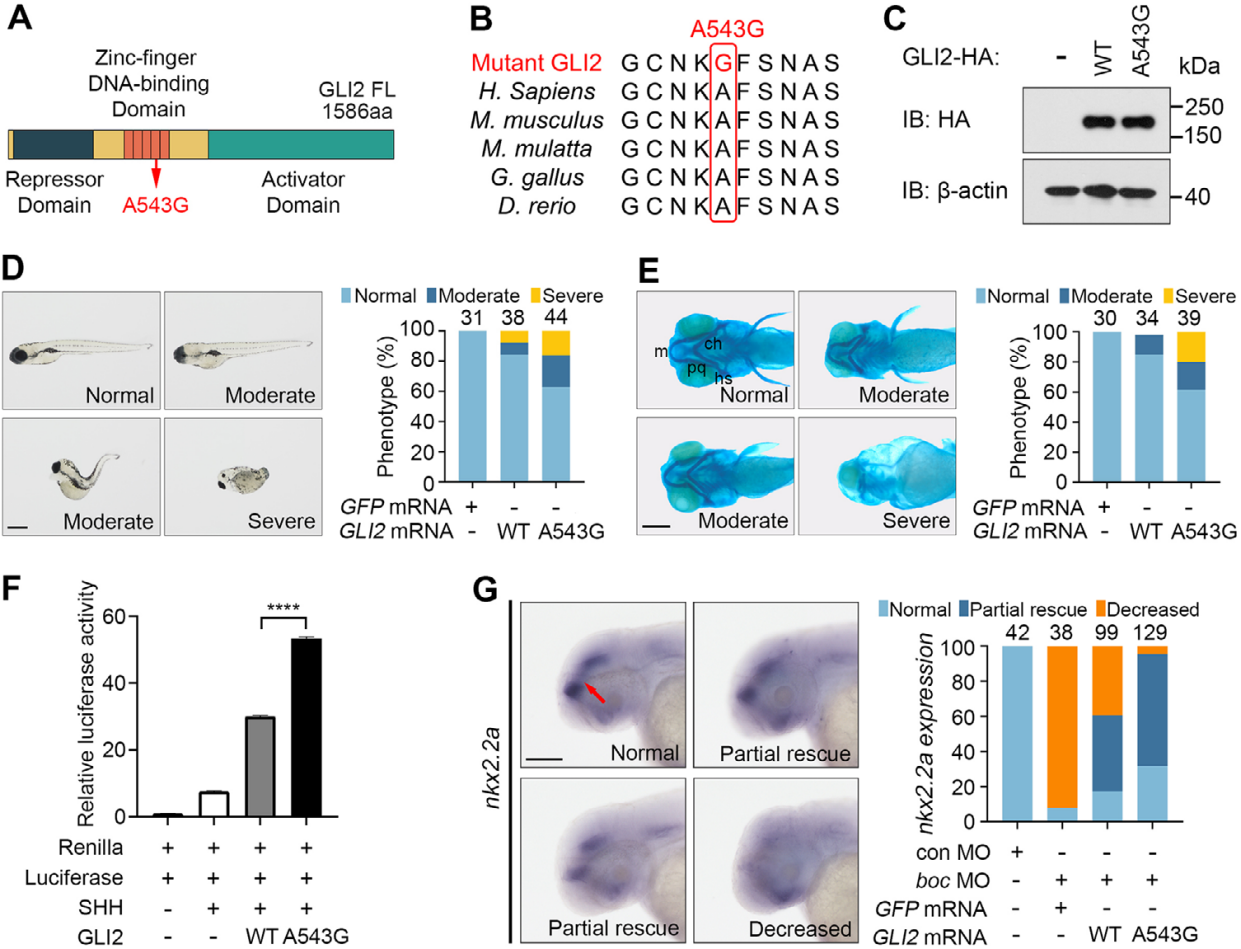
The *GLI2* p.A543G variant acts as a gain‐of‐function mutation in zebrafish embryos. (A) Schematic of GLI2 protein structure showing the Repressor domain, Zinc‐finger DNA‐binding domain, and Activator domain. The location of the amino acid affected by the p.A543G variant is indicated. (B) Multiple sequence alignment showing the conservation of A543 in GLI2 orthologues across vertebrate species. (C) p.A543G variant has no effect on GLI2 protein expression level. HEK293T cells transfected with WT or mutant GLI2 were subjected to Western blot analysis with an HA antibody. β‐actin served as a loading control. (D) p.A543G GLI2 variant is more active in inducing developmental defects in zebrafish embryos. 1‐cell stage zebrafish embryos were injected with 300 pg of mRNAs encoding WT or p.A543G GLI2. Phenotypes were quantified at 5 dpf as in Figure 5A with the same criteria. The number of embryos per condition is indicated on the top of each column. Scale bar, 200 µm. (E) p.A543G GLI2 variant is more potent to induce craniofacial cartilage defects. Zebrafish embryos were microinjected as in (D) and analyzed by Alcian blue staining at 5 dpf. Phenotypes were quantified as in Figure 5B with the same criteria. The number of embryos per condition is indicated on the top of each column. Scale bar, 200 µm. (F) p.A543G GLI2 displays heightened activity in enhancing SHH signaling. NIH3T3 cells were transfected with 8 × 3′Gli‐BS‐delta51‐Luc reporter together with Renilla and WT or p.A543G *GLI2* as indicated. Then the cells were treated as in Figure 6F. Experiments were performed in triplicate. Data are mean ± SD. Statistical significance was assessed by unpaired two‐tailed Student's t‐test. **** *P* < 0.0001. (G) The p.A543G GLI2 variant exhibits enhanced capacity to rescue *nkx2.2a* expression compared to WT GLI2 in *boc* morphants. 1‐cell stage zebrafish embryos were injected with control MO (8 ng) or *boc* MO (8 ng) alone or together with 500 pg of mRNAs encoding either WT GLI2 or the p.A543G GLI2 variant, as indicated. Embryos were analyzed by WISH for *nkx2.2a* expression at 48 hpf. Under normal conditions, *nkx2.2a* is expressed in the forebrain and midbrain, as marked by the red arrow. The number of embryos analyzed per condition is shown at the top of each column. Scale bar: 500 µm.

Our data in both zebrafish embryos and cultured cells demonstrate that the *BOC* p.R681X variant represents the most deleterious loss‐of‐function mutation among the four variants. However, the two affected individuals carrying this variant in the hereditary family only presented with microform cleft lip, the mildest version of CL (Figure 4B). We thus considered whether inheritance at other genetic loci might account for this unusually low expressivity of *BOC* p.R681X variant in the family. We noticed that the proband and her mother, but not her father, also carried a rare missense variant in *GLI2* (c.1628C>G, p.A543G) with low frequencies in examined databases, which was confirmed by Sanger sequencing (Table 1, Figures S5, S6B, and Table S5). GLI2 is a zinc finger transcription factor that acts downstream of *BOC* and translocates to the nucleus to drive target gene expression upon SHH signaling activation.^[^ ^14^, ^18^ ^]^ It consists of an N‐terminal repressor domain, a middle DNA binding domain, and a C‐terminal activator domain.^[^ ^14^, ^18^ ^]^ The altered amino acid Ala543 resides in the DNA binding domain and is invariant in GLI2 orthologues across vertebrate species, indicating a possible functional role of this amino acid (**Figure** **7**A,B).

We surmised that the genetic interaction between *BOC* p.R681X variant and *GLI2* p.A543G variant might account for the mild phenotypes of the carriers and set to evaluate the functional consequence of the *GLI2* p.A543G variant in zebrafish embryos and cultured cells. Western blotting analysis revealed that WT and p.A543G GLI2 were expressed at similar levels, indicating that this variant did not alter protein stability (Figure 7C). 15% of embryos injected with mRNA encoding WT GLI2 exhibited developmental abnormalities similar to those caused by *BOC* overexpression, including curved and shortened body axes and craniofacial defects. In comparison, 36% of embryos displayed these developmental abnormalities upon microinjection of the same dosage of mRNA encoding p.A543G GLI2 variant (Figure 7D). Moreover, Alcian blue staining confirmed that p.A543G GLI2 was twice more active in inducing craniofacial cartilage defects than WT GLI2 (Figure 7E). In keeping with these results, p.A543G GLI2 displayed much‐enhanced activity in further boosting SHH‐stimulated luciferase activity in NIH3T3 cells (Figure 7F). Therefore, the *GLI2* p.A543G variant acts as a hypermorphic mutation in both zebrafish embryos and cultured cells.

To model the genetic interaction between the hypomorphic *BOC* p.R681X variant and the hypermorphic *GLI2* p.A543G variant observed in patients, we established a zebrafish model to recapitulate the human genetic context. Zebrafish embryos were injected with *boc* MO (to mimic p.R681X‐mediated haploinsufficiency) alone or together with mRNAs encoding either WT or p.A543G GLI2. Strikingly, p.A543G GLI2 exhibited significantly enhanced capacity to rescue *boc* MO‐induced *nkx2.2a* downregulation compared to WT GLI2 (Figure. 7G). These results precisely mirror the attenuated clinical phenotype observed in Family 4 patients co‐inheriting the *BOC* p.R681X and *GLI2* p.A543G variants and strongly support an epistatic antagonism between these two variants.

**Figure 8:**
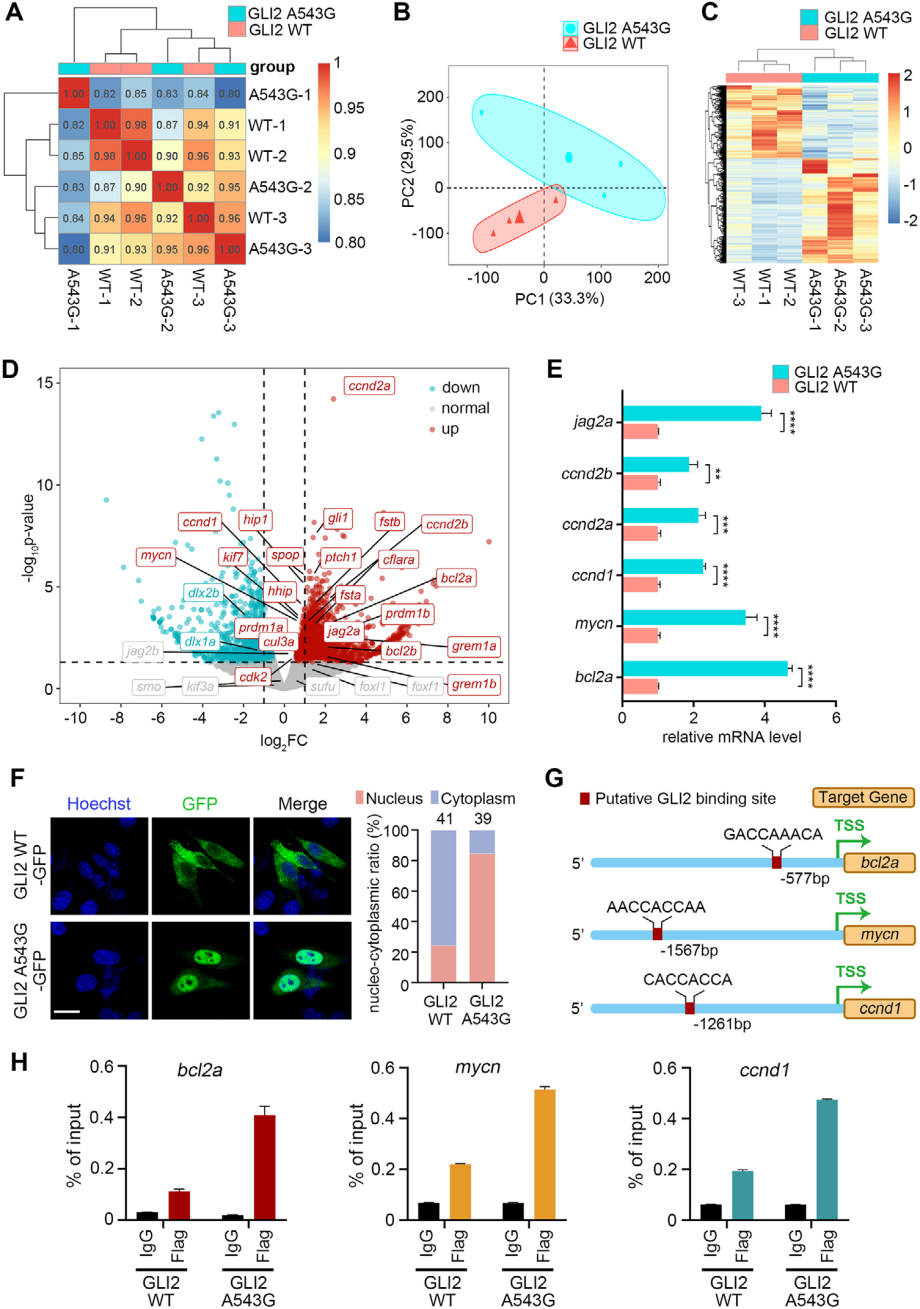
The p.A543G variant enhances GLI2 transcriptional activity by promoting its nuclear localization and increasing its DNA‐binding affinity. (A) Pairwise Spearman rank correlation heatmap illustrating the transcriptional similarity between biological replicates. Each axis represents samples, and each cell shows the pairwise Spearman correlation coefficient. The color gradient indicates correlation strength: red (strong positive correlation) and blue (weak positive correlation). The diagonal (red) denotes perfect self‐correlation. (B) Principal component analysis (PCA) of transcriptomic profiles from zebrafish embryos injected with mRNAs encoding WT (red triangles) or p.A543G (blue circles) GLI2. Each point represents a biological replication. Axes denote the first two principal components (PC1 and PC2), capturing a cumulative 62.8% of the variance in gene expression. Distinct Clustering reflects the separation of transcriptomes based on the expressed WT or p.A543G GLI2. (C) Heatmap displaying hierarchical clustering of differentially expressed genes (DEGs) identified between zebrafish embryos overexpressing WT versus p.A543G GLI2. Color intensity represents row‐wise Z‐score normalized expression levels, with red indicating higher and blue indicating lower relative expression across samples. Both genes (rows) and samples (columns) are clustered based on expression patterns. (D) Volcano plot illustrating differentially expressed genes between zebrafish embryos overexpressing WT versus p.A543G GLI2. Genes are plotted based on log₂ fold change (*x*‐axis) and statistical significance [‐log₁₀(Benjamini‐Hochberg adjusted P‐value), *y*‐axis]. Significantly upregulated (red; adj. *P* ≤ 0.05, log₂FC > 1) and downregulated (blue; adj. *P* ≤ 0.05, log₂FC < ‐1) genes meeting the defined thresholds (dashed lines) are highlighted. (E) Quantitative PCR (qPCR) validation of representative upregulated genes identified by RNA‐seq. Expression levels of *bcl2a*, *mycn*, *ccnd1*, *ccnd2a*, *ccnd2b*, and *jag2a* were significantly elevated in zebrafish embryos overexpressing p.A543G GLI2 than those overexpressing WT GLI2 (Data represent mean ± SEM from *n =* 3 biological replicates). Statistical significance was determined by unpaired two‐tailed Student's t‐test (**P < 0.01, ***P < 0.001, ****P < 0.0001). (F) p.A543G variant promotes GLI2 nuclear localization. Hela cells transfected with GFP‐tagged WT or p.A543G GLI2 were subjected to cellular immunofluorescence analysis. WT GLI2 is primarily expressed in the cytoplasm. Conversely, p.A543G GLI2 is predominantly located within the nucleus. The nucleus was stained with Hoechst (Blue). On the right is the diagram of the proportional subcellular localization distribution of WT and p.A543G GLI2 protein. The number of cells per condition is indicated on the top of each column. Scale bar, 20um. (G) Schematic diagrams showing predicted GLI2 binding motifs (red boxes) in *bcl2a* (‐577 bp), *mycn* (‐1567 bp), and *ccnd1* (‐1261 bp) promoters relative to TSS (transcriptional start site; arrow). All sequences show high similarity to the GACCACCCA consensus motif. These regions were analyzed by ChIP‐qPCR to confirm GLI2 occupancy. (H) The p.A543G GLI2 exhibits enhanced promoter binding affinity. ChIP‐qPCR demonstrates significantly increased binding of the p.A543G GLI2 variant to the promoters of *bcl2a*, *mycn*, and *ccnd1* compared to WT GLI2 in zebrafish embryos at 24 hpf. Embryos were microinjected with mRNA encoding either Flag‐tagged WT or p.A543G GLI2. Results are presented as a percentage of input DNA (mean ± SEM, *n =* 3 biological replicates). IgG was used as the negative control.

To determine how the *GLI2* p.A543G variant exerts its hypermorphic effects, we assessed whether it enhances the transcriptional activity of GLI2. RNA sequencing (RNA‐seq) was performed on zebrafish embryos injected with mRNAs encoding either WT or p.A543G GLI2. Unsupervised clustering analysis revealed distinct expression profiles between these two groups (**Figure** **8**A), and principal component analysis (PCA) further confirmed the clear transcriptomic divergence (PC1: 33.3%, PC2: 29.5%; Figure 8B). Differential expression analysis identified 1921 upregulated genes, including classical targets of SHH signaling pathway, along with 1353 downregulated genes in embryos overexpressing p.A543G GLI2 compared to those overexpressing WT GLI2 (Figure 8C,D). GO and KEGG pathway enrichment analysis revealed overrepresented pathways including muscle development, actin cytoskeleton organization, and HH and TGF‐β signaling—processes critical for craniofacial development (Figure S8). qRT‐PCR validation confirmed elevated expression of SHH signaling pathway targets (*bcl2a*, *mycn*, *ccnd1*, *ccnd2a*, *ccnd2b*, and *jag2a*) in zebrafish embryos overexpressing p.A543G GLI2 relative to those overexpressing WT GLI2 (Figure 8E). Collectively, these findings suggest that the p.A543G variant enhances the transcriptional activity of GLI2, leading to aberrant activation of SHH signaling pathway.

To elucidate the molecular mechanisms driving the enhanced transcriptional activity of p.A543G GLI2, we first examined its subcellular localization. Immunofluorescence assay demonstrated that while most of WT GLI2 was localized in the cytoplasm upon overexpression in Hela cells, the majority of p.A543G GLI2 was in the nucleus, indicating predisposed nuclear localization (Figure 8F). We next tested whether the p.A543G variant increases GLI2 binding to target promoters. Putative GLI2 binding sites were identified in promoters of zebrafish SHH signaling pathway target genes, including *bcl2a*, *mycn*, and *ccnd1* (Figure 8G). As expected, chromatin immunoprecipitation followed by qPCR (ChIP‐qPCR) revealed significantly higher occupancy of p.A543G GLI2 at these loci compared to WT GLI2 (Figure 8H). Together, these results demonstrate that the p.A543G variant potentiates GLI2 transcriptional activity by synergistically promoting its nuclear localization and increasing its binding to target promoters.

Taken together, these results indicate that *GLI2* p.A543G variant acts as a gain‐of‐function mutation both in vivo and in vitro and illustrate a model of two‐locus transmission of cleft lip in a hereditary family, whereby a hypermorphic mutation in *GLI2* counteracts a hypomorphic mutation in *BOC*, giving rise to the unexpected microform cleft lip phenotype.

## 3 Discussion

Our study suggests three main conclusions. First, we identify many candidate variants for NSOFCs by ES in 214 sporadic cases, which deepens our understanding of the genetic architecture of NSOFCs and provides a fruitful resource for future research. Second, we provide both in vitro and in vivo evidence that *BOC* is a novel causal gene for NSOFCs. Third, we find an unusual co‐inheritance of a loss‐of‐function mutation in *BOC* and a gain‐of‐function mutation in *GLI2* in a multiplex family and demonstrate that the epistatic antagonism of them accounts for the mild phenotype in affected individuals, which represents a previously undiscovered model of inheritance for OFCs.

We recruited 214 sporadic cases from the Han Chinese population and conducted ES to delineate the genetic architecture of NSOFCs. Using a gene panel comprised of recently published 418 OFC genes and additional 154 OFC‐related signaling pathway genes for variants filtering, we were able to identify many candidate variants, including 127 P/LP variants in 80 genes and 642 VUS in 302 genes. Interestingly, 11 of the 80 genes harboring P/LP variants were not included in the 418 OFC genes and thus represented new OFC genes, which, together with *BOC* gene validated by our functional experiments, provided an updated version of 430 OFC genes (Figure S9). While the high ratio of recurrently mutated genes demonstrates allelic heterogeneity of NSOFCs and the discrepancy in underpinning mutated genes across OFC subtypes supports the long‐hold notion of extensive genetic heterogeneity in NSOFCs, the presence of genetic overlap is also unambiguous. Our findings also highlight the critical roles of perturbation of OFC‐related signaling pathways and their crosstalk in the pathogenesis of NSOFCs and lend credence to our proposition that genetic variants, including both P/LP variants and VUS, that might affect OFC‐related signaling pathways are more likely to be pathogenic mutations and should be prioritized for further functional validation.

**Figure 9:**
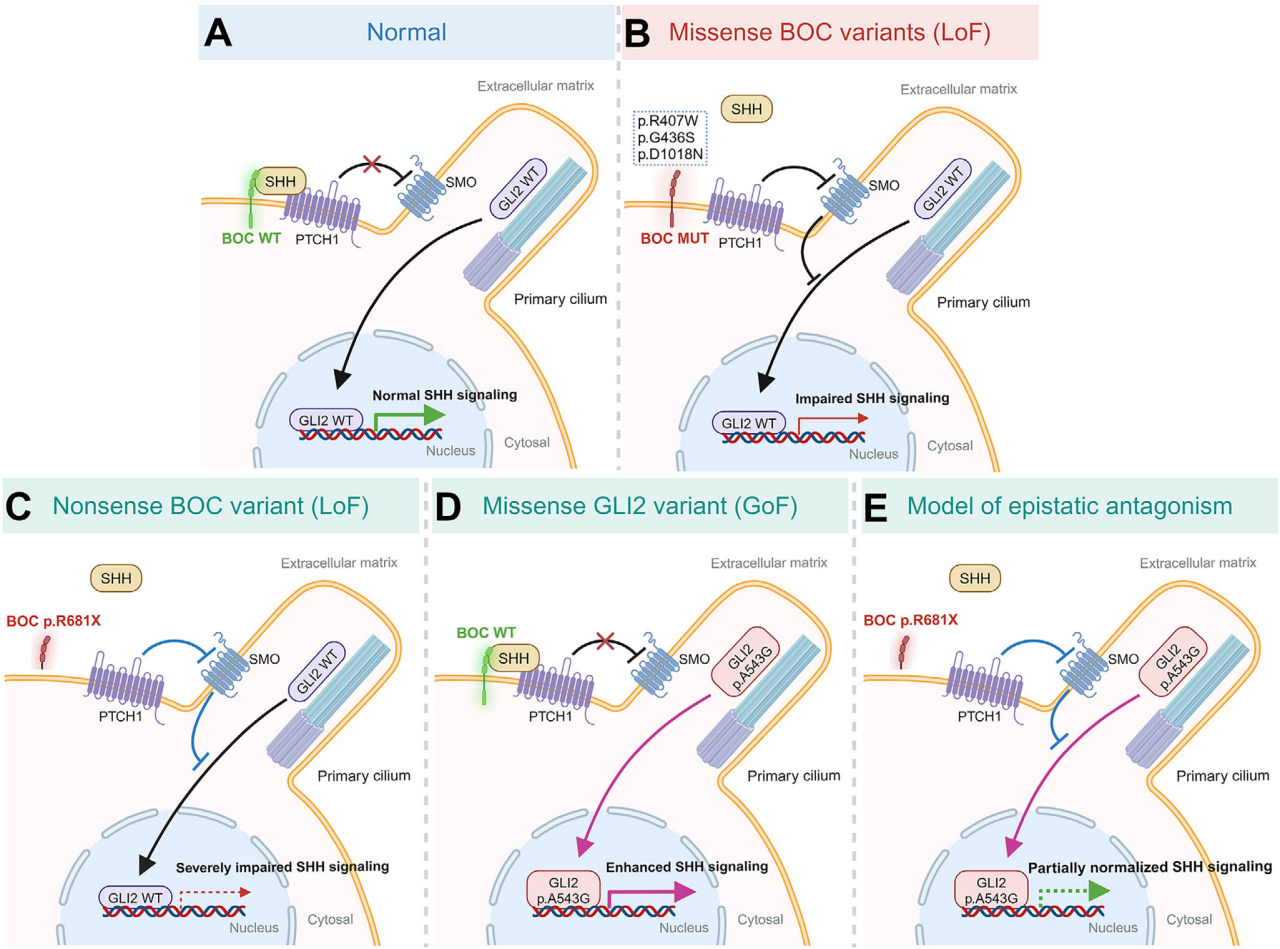
Schematic diagrams illustrating the pathogenic mechanism of *BOC* missense variants and the epistatic antagonism between the hypomorphic *BOC* p.R681X variant and the hypermorphic *GLI2* p.A543G variant. (A) Under normal physiological conditions, *BOC* acts as a co‐receptor for SHH. SHH binding to PTCH1 and *BOC* leads to the de‐repression of SMO, which in turn activates signal transduction events that result in GLI2‐mediated transcriptional activation. (B) *BOC* missense variants (p.R407W, p.G436S, and p.D1018N) disrupt *BOC* membrane localization and/or its binding to SHH and PTCH1, therefore inhibiting GLI2‐mediated transcriptional activation. (C) The p.R681X *BOC* is secreted to extracellular space and loses SHH binding activity, resulting in haploinsufficiency of *BOC* and severely impaired GLI2‐mediated transcriptional activation. (D) The *GLI2* p.A543G variant results in boosted GLI2‐mediated transcriptional activation. (E) Epistatic antagonism between the hypomorphic *BOC* p.R681X variant and hypermorphic *GLI2* p.A543G variant, which partially normalizes SHH signaling and accounts for the mild phenotype in the proband and affected her mother in the multiplex family.

We identify *BOC*, which encodes a SHH signaling co‐receptor, as a novel causal gene for NSOFCs. Disruption of SHH signaling in humans is best known to cause holoprosencephaly (HPE), a condition arising from defective patterning of the forebrain and midface and encompassing a wide phenotypic spectrum ranging from failure to partition the forebrain into hemispheres and cyclopia, to mild midfacial deformities without forebrain involvement.^[^ ^19^ ^]^ Although mice lacking Boc do not show HPE, loss of Boc in a *Cdon* or *Gas1* null background does exacerbate their HPE phenotype, highlighting its role as a genetic modifier in HPE.^[^ ^20^ ^]^ Consistent with this, several hypomorphic *BOC* missense variants have been associated with HPE in humans.^[^ ^21^ ^]^ Here, we demonstrate that both missense and nonsense *BOC* variants act as causative mutations for NSOFCs (**Figure** **9**A–C), extending their pathogenic role beyond HPE. The variable expressivity observed in HPE and OFCs^[^ ^20^, ^22^ ^]^ suggests that *BOC* deficiency may underlie a broader spectrum of craniofacial defects with unresolved genetic etiologies. Clinically, incorporating *BOC* into diagnostic panels could resolve a subset of idiopathic OFC cases, particularly those with subtle midline dysmorphology or familial recurrence. Therapeutically, small‐molecule SHH agonists (e.g., SAG)^[^ ^23^ ^]^ may prevent craniofacial defects in high‐risk *BOC* variant carriers by normalizing impaired SHH signaling. Together, this work repositions *BOC* from an HPE modifier to a primary NSOFC gene, offering new avenues for molecular diagnosis and targeted intervention in craniofacial disorders.

Genetic interactions between risk alleles play a pivotal role in OFC pathogenesis, influencing both syndromic and non‐syndromic forms.^[^ ^24^ ^]^ In Family 4, we identified a novel epistatic interaction between two functionally antagonistic variants: a hypomorphic *BOC* variant (p.R681X) and a hypermorphic *GLI2* variant (p.A543G). The *BOC* p.R681X nonsense mutation abolishes SHH binding due to defective protein localization, causing haploinsufficiency and impairing SHH signal transduction. In contrast, the *GLI2* p.A543G mutation, located in its DNA‐binding domain, enhances GLI2 transcriptional activity via increased nuclear retention and DNA‐binding affinity, leading to hyperactive SHH signaling. Strikingly, co‐inheritance of these variants yields a compensatory interaction: GLI2 hyperactivity partially rescues the SHH signaling deficit caused by *BOC* haploinsufficiency, restoring transcriptional output to near‐physiological levels. Therefore, these findings implicate a two‐locus model of inheritance in NSOFCs via epistatic antagonism between a rare hypomorphic *BOC* allele and a rare hypermorphic *GLI2* allele, which provides an explanation for the microform cleft lip in the carriers (Figure 9C–E). While the evidence implicating *BOC* in cleft palate pathogenesis is compelling, the putative epistatic role of *GLI2* p.A543G variant, despite being supported by comprehensive functional analyses, warrants further validation given its reliance on a single familial cohort, thus precluding its immediate application in diagnostic or prenatal settings. Nevertheless, this mode of inheritance, first discovered here to the best of our knowledge, is distinct from those in previous studies of genetic interactions between risk alleles (e.g., digenic inheritance) in NSOFCs in which variants in different loci are likely to have synergistic effects on the phenotype and might account for a portion of OFCs where genotype‐phenotype inconsistency cannot be explained by incomplete penetrance or variable expressivity. This genetic paradigm of compensatory variant interactions may represent a broader mechanism that also underlies the pathogenesis of other complex developmental disorders.

While the present study was conducted exclusively in a Han Chinese population from Western China, the fundamental gene regulatory networks governing lip and palate development and NSOFC pathogenesis are known to exhibit high evolutionary conservation across human populations,^[^ ^2b^ ^]^ suggesting that our findings may have broader biological implications beyond the studied cohort. However, the allele frequencies and effect sizes of identified variants may differ across populations due to genetic diversity.^[^ ^25^ ^]^ Additionally, population‐specific differences in environmental risk factors (e.g., maternal nutrition status, teratogen exposure, or healthcare access) can influence disease susceptibility. These genetic and environmental heterogeneities may limit the direct generalizability of our findings to other cohorts. To address these limitations, future studies should prioritize systematic validation in ethnically diverse cohorts, employ multi‐ethnic meta‐analytical frameworks to disentangle genetic and environmental contributions and integrate large‐scale trans‐ancestral datasets to refine etiological models of NSOFCs.

Clinically, prenatal ultrasound screening has significantly reduced the incidence of severe OFCs (both syndromic and non‐syndromic forms) over the last two decades,^[^ ^26^ ^]^ but it still has difficulty in detecting CP and some OFCs with subclinical phenotypes. The widespread implementation of GWAS, ES, and WGS are enabling us to gain a better understanding of the genetic etiology of OFCs. The novel variants identified in our study expand existing OFC gene panels, enhancing clinical genetic testing capabilities. Incorporating these findings into diagnostic workflows can significantly improve molecular diagnosis accuracy, recurrence risk prediction, and reproductive counseling for affected families. Furthermore, these genetic markers may prove particularly valuable in cases where prenatal ultrasound yields inconclusive results, offering a complementary diagnostic approach when structural assessment is insufficient.

## 4 Experimental Section

### Clinical Samples

Over the past four years, 214 unrelated individuals presenting with sporadic NSOFCs (104 males and 110 females; 81 CL cases, 111 CP cases, and 22 CLP cases) were enrolled, alongside a control group of 102 unaffected individuals. All participants in this study were of Han Chinese descent. Comprehensive examinations of both patients and control subjects were conducted by two experienced surgeons to ensure precise phenotypic characterization of craniofacial anomalies and other related organic manifestations. Genomic DNA was extracted from peripheral blood samples using the QIAamp DNA Blood Mini Kit (Qiagen, #51106).

### Exome Sequencing

Exome sequencing (ES) was conducted on 214 sporadic cases affected by NSOFCs, comprising two cohorts: Patient Cohort 1 (138 patients) and Patient Cohort 2 (76 patients). ES for Patient Cohort 1 was conducted using the BGISEQ‐500 platform (BGI Inc., China), while the Illumina PE150 platform (Novogene Co., Ltd., China) was used for Patient Cohort 2. Raw data were filtered to obtain clean data using the following criteria: 1) read pairs containing adapters were removed; 2) read pairs with more than 10% “N” bases in a single‐end read were discarded; and 3) read pairs with over 50% low‐quality bases (below a quality score of 5) in a single‐end read were excluded. After quality control, the clean data from both cohorts were mapped to the GRCh37/hg19 reference genome using Burrows‐Wheeler Aligner software. Duplicate reads were marked and removed using the Picard tool (http://broadinstitute.github.io/picard/) for Patient Cohort 1 and the Sambamba tool for Patient Cohort 2. Then, variant calling was conducted using HaplotypeCaller for Patient Cohort 1 and SAMtools for Patient Cohort 2. Finally, variant annotation was conducted using SnpEff for Patient Cohort 1 and ANNOVAR for Patient Cohort 2, resulting in the identification of SNV and INDEL variants.

To identify potential pathogenic variants in these 214 sporadic NSOFC patients, common variants were first filtered out using population genomic databases. Following the criteria established in our prior work, variants were excluded with a minor allele frequency > 0.5%.^[^ ^27^ ^]^ And subsequently focused on variants predicted to alter protein function (missense, nonsense, insertion/deletion, and splice‐site variants) due to their higher likelihood of deleterious effects compared to non‐coding variants.^[^ ^8d^ ^]^ To prioritize variants potentially associated with OFCs, a targeted gene panel comprising: 1) 418 published OFC‐associated genes,^[^ ^9^ ^]^ and 2) 154 genes encoding critical components of developmental signaling pathways (SHH, FGF, WNT, and TGF‐β/BMP) involved in lip and palate development and OFC pathogenesis (Table S1) were then employed.^[^ ^4^ ^]^ Finally, variant pathogenicity was classified according to ACMG guidelines,^[^ ^28^ ^]^ using VarSome and Franklin for automated annotation with manual review.^[^ ^29^ ^]^ Our pipeline for identifying candidate causal genes and variants in the multiplex family followed our previously established methodology.^[^ ^8e^ ^]^

To validate the variants detected through ES, PCR‐Sanger sequencing was employed using specifically designed primers. For any candidate variant previously not documented in genomic databases, the validation process was extended to include PCR‐Sanger sequencing in a control cohort comprising 102 healthy individuals. This additional step was undertaken to confirm the rarity of these variants, thereby substantiating their potential role in the pathogenesis of NSOFCs.

### Cloning

Full‐length human *BOC* (NM_001301861), *SHH* (NM_001310462.2), and *GLI2* (NM_005270) were cloned into pCS2 vectors with carboxyl‐terminal HA or Flag tags. Human *GLI2* (NM_005270) was also cloned into pEGFP‐N1 vector. Variants of *BOC* and *GLI2* were generated through site‐directed mutagenesis. The coding sequences of the extracellular domain (ECD) of WT or mutant *BOC* (amino acids 1–855) were cloned into pCS2 vectors with a carboxyl‐terminal HA tag. All constructs were confirmed by Sanger sequencing.

### Cell Culture and Transfection

HEK293T, Hela, and NIH3T3 cells were cultured in DMEM medium supplemented with 10% fetal bovine serum (Omega, #FB‐01) and 1% penicillin‐streptomycin, in a 5% CO_2_ incubator at 37 °C. All cell lines were transfected with Lipo3000 reagent (Thermo, #L3000015) following the manufacturer's instructions.

### Secretion Assay

HEK293T cells were transfected with pCS2‐SHH‐Flag construct. 24 h later, the cells were washed with PBS twice to remove residue serum, and the culture medium was replaced with serum‐free DMEM. 30 h later, conditioned media were collected, centrifuged to remove cell debris, and concentrated five times using Amicon Ultra centrifugal filter‐10K (Millipore, cat#UFC501024) following manufacturer's indications and analyzed by Western blotting.

### Immunoprecipitation, Western Blotting and Immunofluorescence

Immunoprecipitation (IP), Western blotting, and Immunofluorescence (IF) were performed as described previously.^[^ ^30^ ^]^ The antibodies used were PTCH1 (CST, #2468S), HA (Proteintech, # 51064‐2‐AP), FLAG (Origene, #TA180144) and β‐actin (Origene, #TA811000).

### Pull‐Down Assay

Plasmids encoding SHH‐Flag and the extracellular domain (ECD) of WT or mutant *BOC* were transfected into HEK293T cells. 24 h post‐transfection, the cells were cultured for an additional 36 h in serum‐free DMEM. Subsequently, the culture media was collected and an equal amount of medium containing SHH‐Flag was incubated with media containing ECD of WT or mutant *BOC* overnight at 4 °C with an anti‐FLAG antibody (Santa Cruz, #sc‐166355) and protein A/G PLUS‐Agarose beads (Santa Cruz, #sc‐2003). The beads were washed with PBS containing 0.1% NP40 for five times and heated for 5 min at 95 °C in 50 µL of 2× Laemmli buffer to elute protein complexes, followed by analysis through SDS‐PAGE and Western blotting.

### Luciferase Reporter Assay

NIH3T3 cells were transfected with 8 × 3′Gli‐BS‐delta51‐Luc reporter together with pRL‐TK Renilla and various constructs as indicated in corresponding figure legends. 24 h after transfection, cells were serum starved for 12 h and then stimulated with 100 ng mL^−1^ SHH protein (R&D systems, #1845‐SH) for 16 h. Cells were lysed in Passive Lysis Buffer (Promega) and Luciferase assays were performed with the Dual‐Luciferase Reporter Assay System (Promega, #E1980) according to manufacturer's instructions, using a Glomax Luminometer (Promega). Renilla readings were used for normalization.^[^ ^30a^ ^]^

### Zebrafish

AB strains purchased from the China Zebrafish Resource Center (CZRC) were used. Embryos were raised at 28.5 °C and staged as described.^[^ ^31^ ^]^ Fish maintenance followed the Institutional Animal Care and Use Committee (IACUC) protocol.

### mRNA Synthesis, Morpholino and Microinjection

WT or mutant forms of pCS2 *BOC*‐HA and pCS2 GLI2‐HA were linearized with Not1 and transcribed using the mMESSAGE mMACHINETM SP6 Transcription Kit (Invitrogen, #AM1340). mRNA was microinjected into zebrafish embryos at the one‐cell stage and the embryos were cultured till 5 days post‐fertilization (dpf), as described previously.^[^ ^30a^ ^]^ The translation‐blocking *boc* morpholino (*boc* MO: 5′‐AATCCAATTCAACGTCCCAGACATC‐3′) has been described previously.^[^ ^17b^ ^]^

### Alcian Blue Staining and In Situ Hybridization

Zebrafish embryos were cultured until 5 dpf and subsequently fixed overnight in 4% paraformaldehyde (PFA). Then the embryos were stained using a 0.1 mg mL^−1^ Alcian blue reagent (Sigma, #5268). After destaining, the embryos were further fixed using glycerin and photographed using a ZEISS Axio Zoom V16 microscope, following the protocol described in our previous study.^[^ ^30a^ ^]^ In situ hybridization was performed according to a standard protocol with *col2a1* or *nkx.2.2a* probes.^[^ ^32^ ^]^

### RNA‐sequencing

Zebrafish embryos (AB strain) at the 1‐cell stage were microinjected with 400 pg of mRNAs encoding either WT or p.A543G GLI2 and cultured until 24 hpf. Total RNA was extracted from 100‐embryo pools (*n =* 3 replicates per group) using the miniBEST Universal RNA Extraction Kit (TaKaRa, #9767), with quality verified by Bioanalyzer, Qubit quantification, and OD_260/280_ ratios. Stranded mRNA‐seq libraries were prepared and sequenced on an Illumina NovaSeq 6000 (150 bp paired‐end) platform in Novogene Bioinformatics Technology (Beijing, China). Reads were aligned to the zebrafish genome (ensembl_109_danio_rerio_grcz11_primary). Differentially expressed genes (DEGs) were identified using edgeR with thresholds of |log₂FC| > 1 (2‐fold change) and false discovery rate (FDR) < 0.05. The RNA‐sequencing data generated in this study have been deposited in the Gene Expression Omnibus (GEO) database under accession number GSE292867.

### Quantitative PCR

Complementary DNA (cDNA) was synthesized from 1 µg of total RNA isolated from zebrafish embryos using the cDNA synthesis kit (Accurate Biotechnology, # AG11728) according to the manufacturer's protocol. Quantitative real‐time PCR was conducted using the Quant Gene 9600 system (Hangzhou Bioer Technology) with SYBR Green Premix (Accurate Biotechnology, #AG11701). Relative gene expression levels were normalized to *tbp* (TATA‐box binding protein) and calculated using the 2^(‐ΔΔCt) method. Gene‐specific primer sequences (designed to span exon‐exon junctions where applicable) are provided in Supplementary Table S5.

### GO and KEGG Enrichment Analysis

To characterize the various gene sets, gene ontology (GO) and KEGG pathway enrichment analysis were performed using the clusterProfiler platform.^[^ ^11^ ^]^

### Chromatin Immunoprecipitation (ChIP) Assay

Chromatin immunoprecipitation (ChIP) was performed following an established protocol^[^ ^33^ ^]^ using the ChIP Kit (Thermo Fisher Scientific, Cat#26156) according to the manufacturer's instructions. Zebrafish embryos (AB strain) at the single‐cell stage were microinjected with 400 pg of mRNAs encoding Flag‐tagged WT or p.A543G GLI2 and cultured until 24 hpf. For each experimental group (WT or A543G), >1000 zebrafish larvae were collected and mechanically dissociated using a 21‐gauge needle, followed by cross‐linking with 1% formaldehyde (Thermo Fisher Scientific, #28906) for 10 min at room temperature. Chromatin was extracted using lysis buffer and fragmented to 200–800 bp using Micrococcal Nuclease (MNase). Immunoprecipitation was performed overnight at 4 °C using an anti‐FLAG antibody (Sigma‐Aldrich, #F3165) or mouse IgG (Santa Cruz, #sc‐2025) as a negative control. Purified DNA was isolated using the kit's DNA Clean‐Up Columns and analyzed by quantitative PCR (qPCR). To identify putative GLI2‐binding sites in the promoters of target genes, in silico prediction was performed using the NovoPro online tool (https://www.novopro.cn/tools/fuzzy_search_dna.html). Primer sequences for qPCR amplification are listed in Table S5.

### Animal Ethics

All zebrafish experiments of this study were approved by the Committee on the Ethics of Animal Experiments of Xi'an Jiaotong University (No.XJTULAC2020‐411) and performed in strict accordance with the animal care and use guidelines.

### Human Ethics

This work was approved by the Ethics Committee at the Hospital of Stomatology, Xi'an Jiaotong University (xjkqll[2019]NO.014), and informed consent was obtained from the participants or their guardians.

### Statistical Analyses

Statistical significance was assessed by an unpaired two‐tailed Student's t‐test in SHH reporter assay. Statistically significant results in all figures are indicated as *: *P*< 0.05; **: *P*< 0.01; ***: *P*< 0.001; and ****: *P*< 0.0001.

## Conflict of Interest

The authors declare that they have no conflict of interest.

## Author Contributions

Q.H., M.Y., and Y.J. contributed equally to this work. Y.D., H.Z., C.Y., and H.H. designed research and supervised the project; Q.H., M.Y., Y.X., Y.J., X.L., W.H., L.X., Y. H., Z.R., H.Z., B.L., Z.Q., P.L., J.Z., and Y.D. performed research; Y.D., H.Z., C.Y., and H.H. analyzed data; Y.D., Q.H., H.H., C.Y., and H.Z. wrote the paper.

## Supporting information



Supporting Information

Supporting Information

Supporting Information

Supporting Information

Supporting Information

Supporting Information

## Data Availability

The data that support the findings of this study are available in the supplementary material of this article.
