# Supplementary material for: Exome Sequencing Reveals the Genetic Architecture of Non‐syndromic Orofacial Clefts and Identifies BOC as a Novel Causal Gene
Source: Adv Sci (Weinh). 2025 Jun 4;12(32):e12073. doi: 10.1002/advs.202412073 (PMC12407381; doi:10.1002/advs.202412073)
Supplement: Supplementary file 1 — Supporting Information [file ADVS-12-e12073-s001.docx]

**Supplemental Information for**

**Exome sequencing reveals the genetic architecture of non-syndromic orofacial clefts and identifies *BOC* as a novel causal gene**

Qing He^a,b,1^, Min Yu^c,1^, Yuhua Jiao^b,d,1^, Yizhu Xu^b,d^, Xuqin Liang^a^, Wenbin Huang^e^, Linping Xu^a^, Yuxia Hou^b,d^, Zhanping Ren^b,f^, Beile Lyu^g^, Zhenwei Qian^h^, Pengpeng Liu^i^, Jing Zhou^j^, Huimei Huang^k,2^, Chunyan Yin^j,2^, Huaxiang Zhao^b,d,2^, and Yi Ding ^a,j,2^

^1^equal contribution

^2^To whom correspondence should be addressed. Email: hmhuang2000@163.com (H.H.); yinchunyan@mail.xjtu.edu.cn (C.Y.); huaxiangzhao@xjtu.edu.cn (H.Z.); dingyi1510@xjtu.edu.cn (Y.D.).

**Supplemental Information includes:** SI Figures S1-S9

**
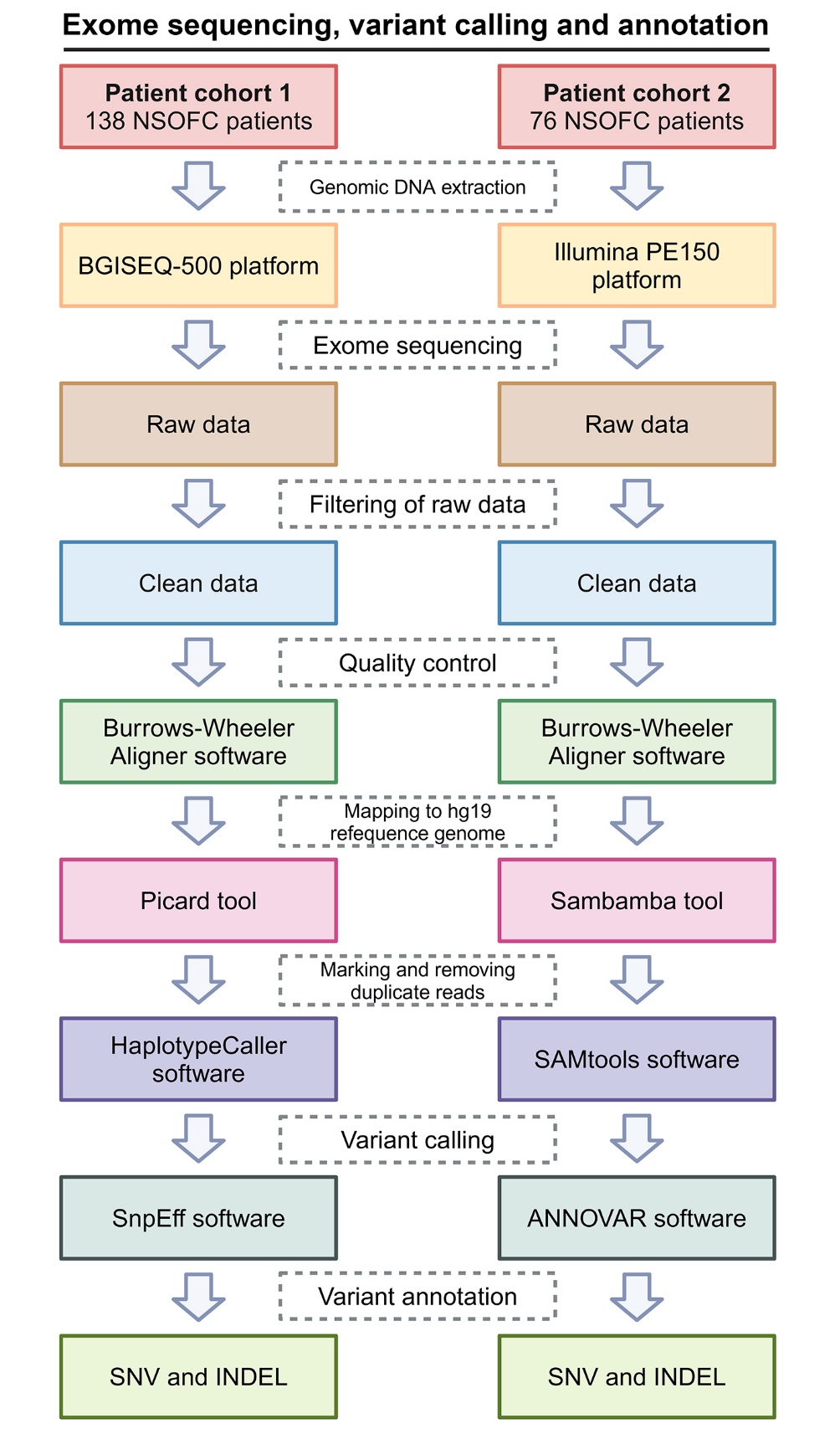
**

**Figure S1. Workflow diagram of the exome sequencing data processing, including variant calling and annotation, for 214 sporadic NSOFC patients.
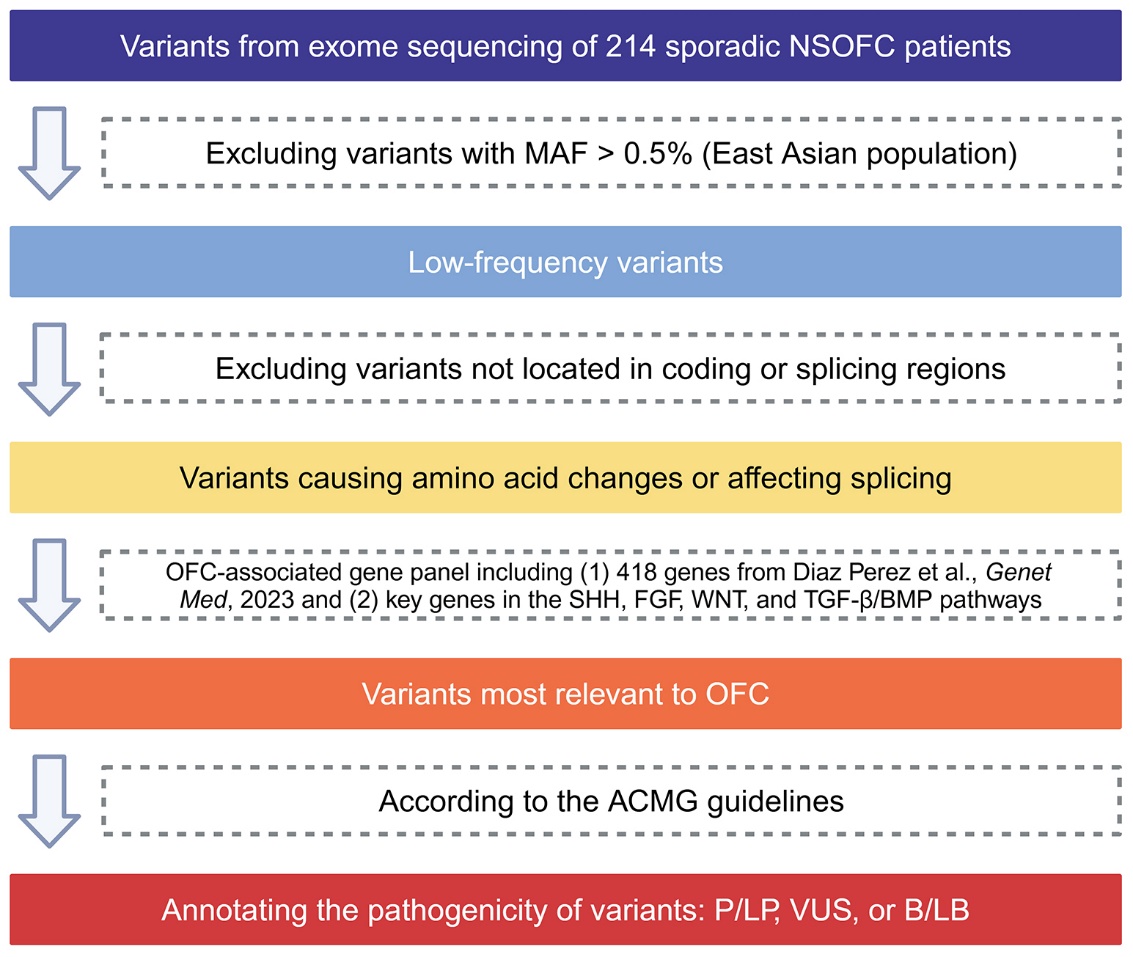
**

**Figure S2. A flowchart depicting the filtering and prioritization of candidate pathogenic variants in 214 sporadic NSOFC patients.**

**
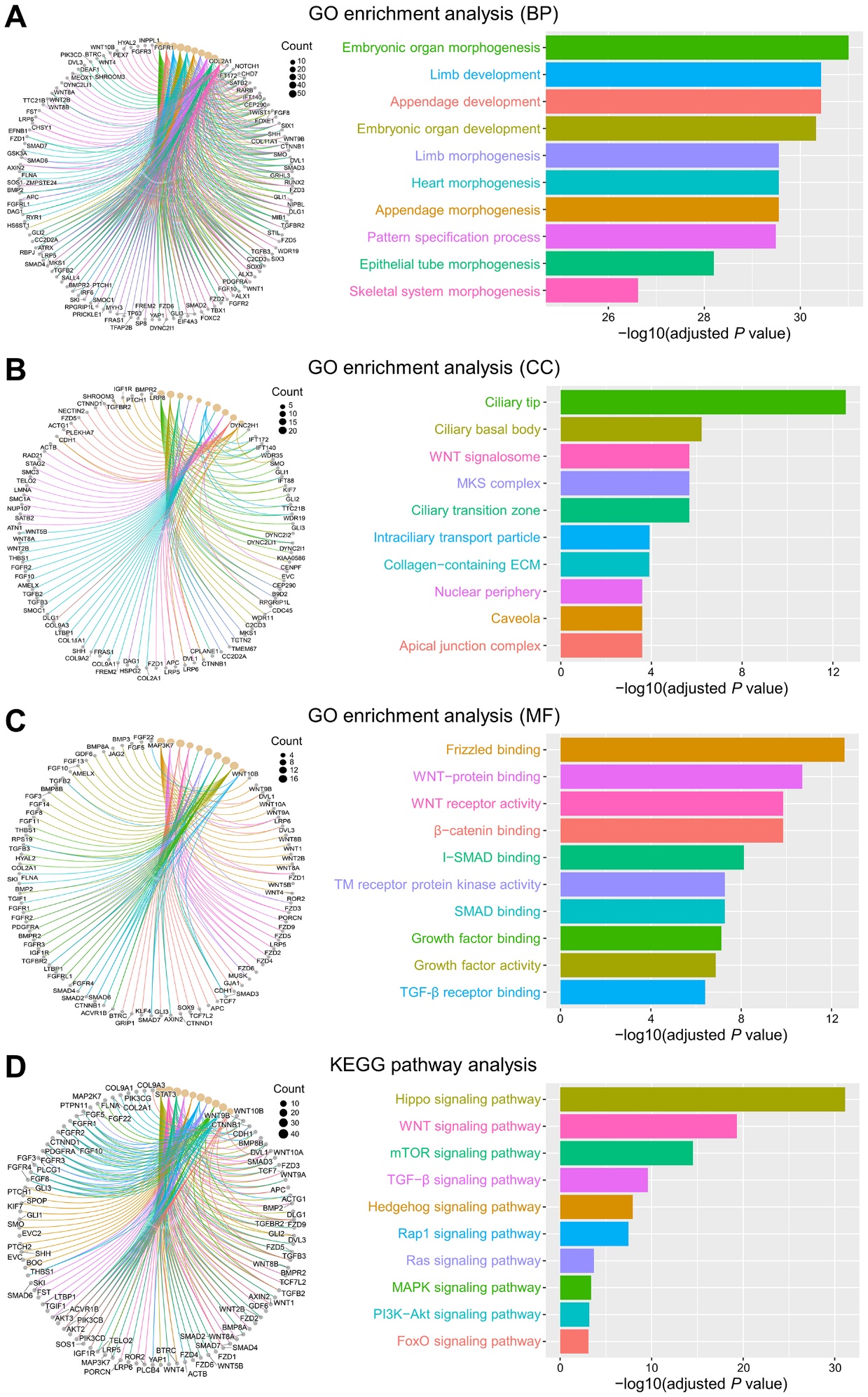
**

**Figure S3. GO and KEGG pathway enrichment analysis of the candidate genes harboring P/LP variants and VUS.** (A) GO- biological process. (B) GO-cellular components. (C) GO-molecular function. (D) KEGG pathway. The size of the circles represents the count of genes in each enriched term in the left panel. The color of the different lines on the left panel and bars on the right panel represent the different enriched terms.

**
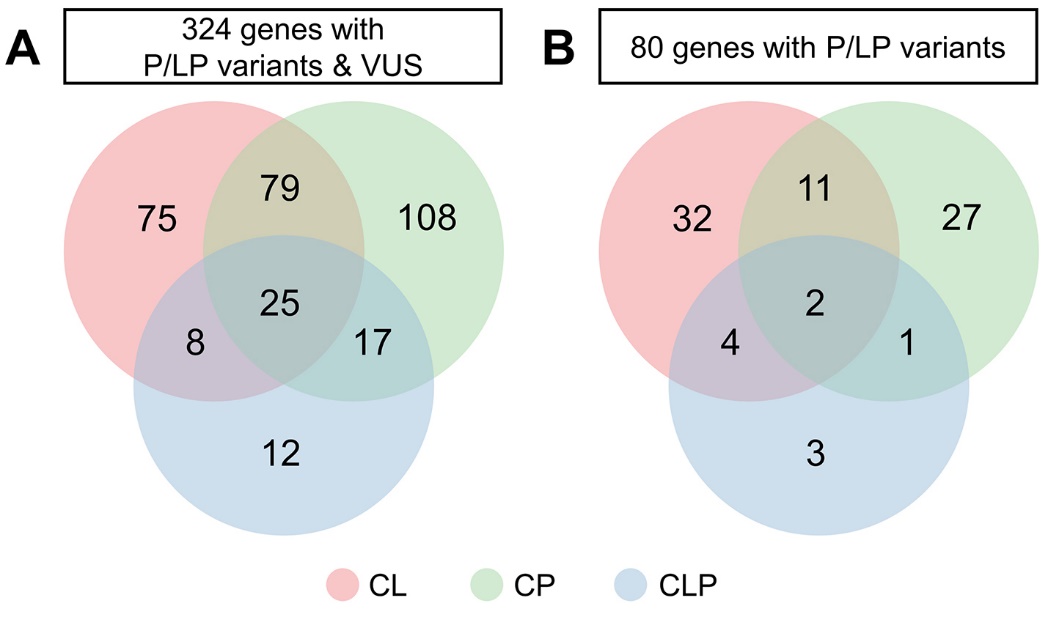
**

**Figure S4. Genetic overlap between OFC subtypes.** Venn diagram showing overlap of genes with P/LP variants and VUS (A) or P/LP variants only (B) between OFC subtypes.

**
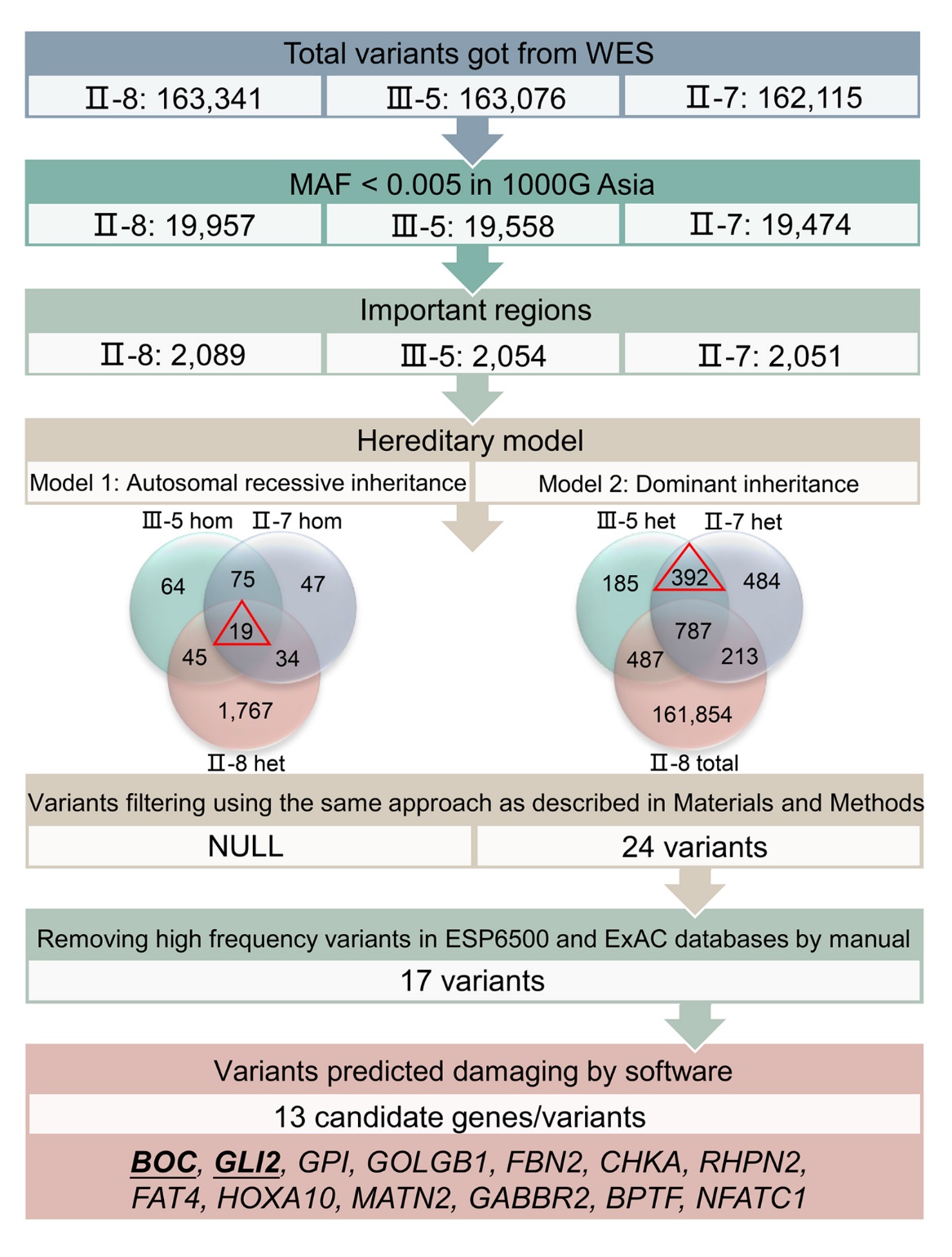
**

**Figure S5. Flowchart for screening candidate rare variants in the multiplex family with NSOFCs.** Note that the proband and her affected mother carried variants in both *BOC* and *GLI2*.

**
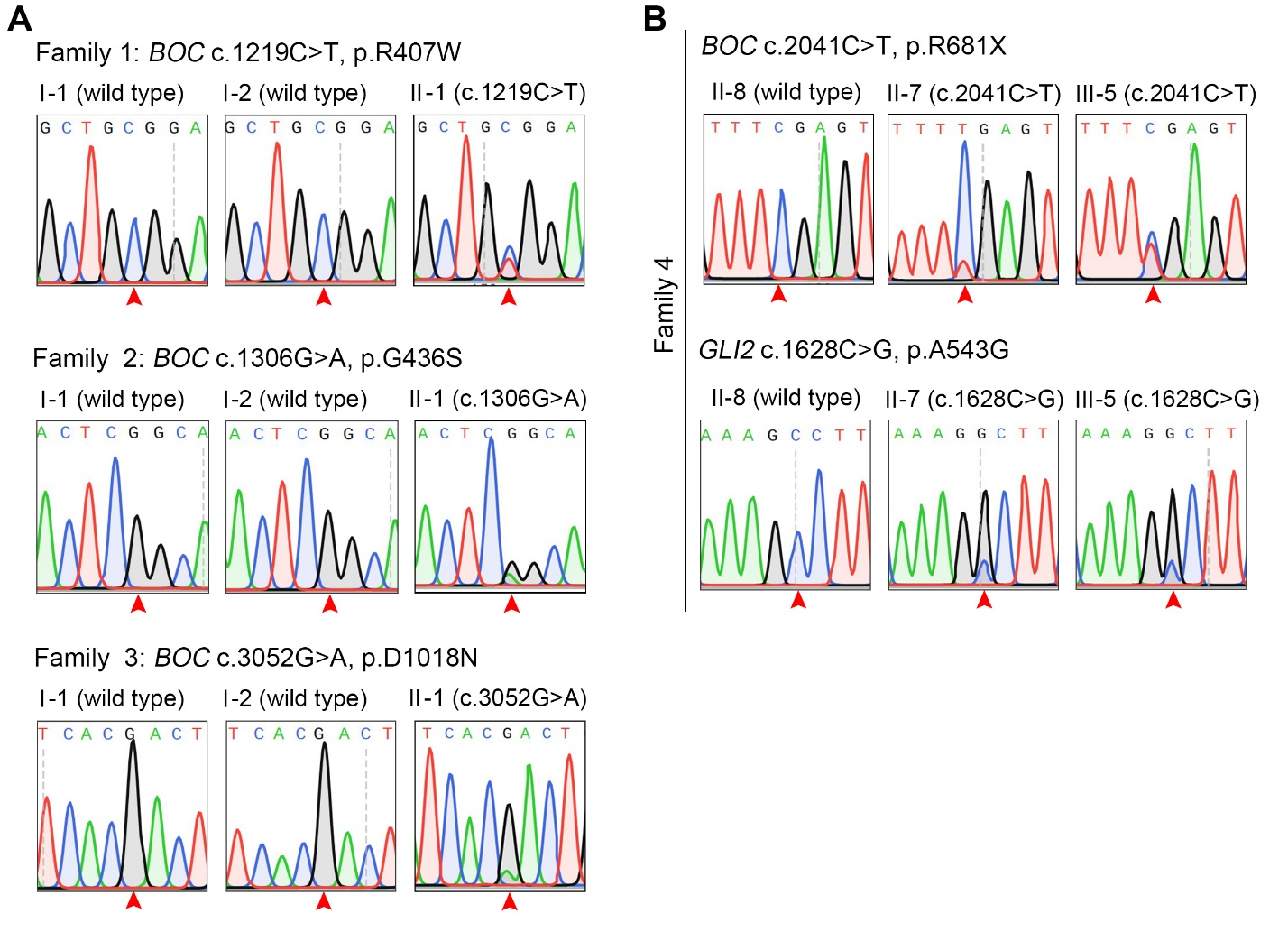
**

**Figure S6. Sanger sequencing chromatograms of *BOC* and *GLI2* variants in NSOFC patients.** (A) Sanger sequencing chromatograms of the three heterozygous *BOC* missense variants (c.1219C>T, p.R407W; c.1306G>A, p.G436S; c.3052G>A, p.D1018N) in Families 1-3. (B) Sanger sequencing chromatograms of the heterozygous *BOC* nonsense variant (c.2041C>T, p.R681X) and the heterozygous *GLI2* missense variant (c.1628C>G, p.A543G) in Family 4.

**
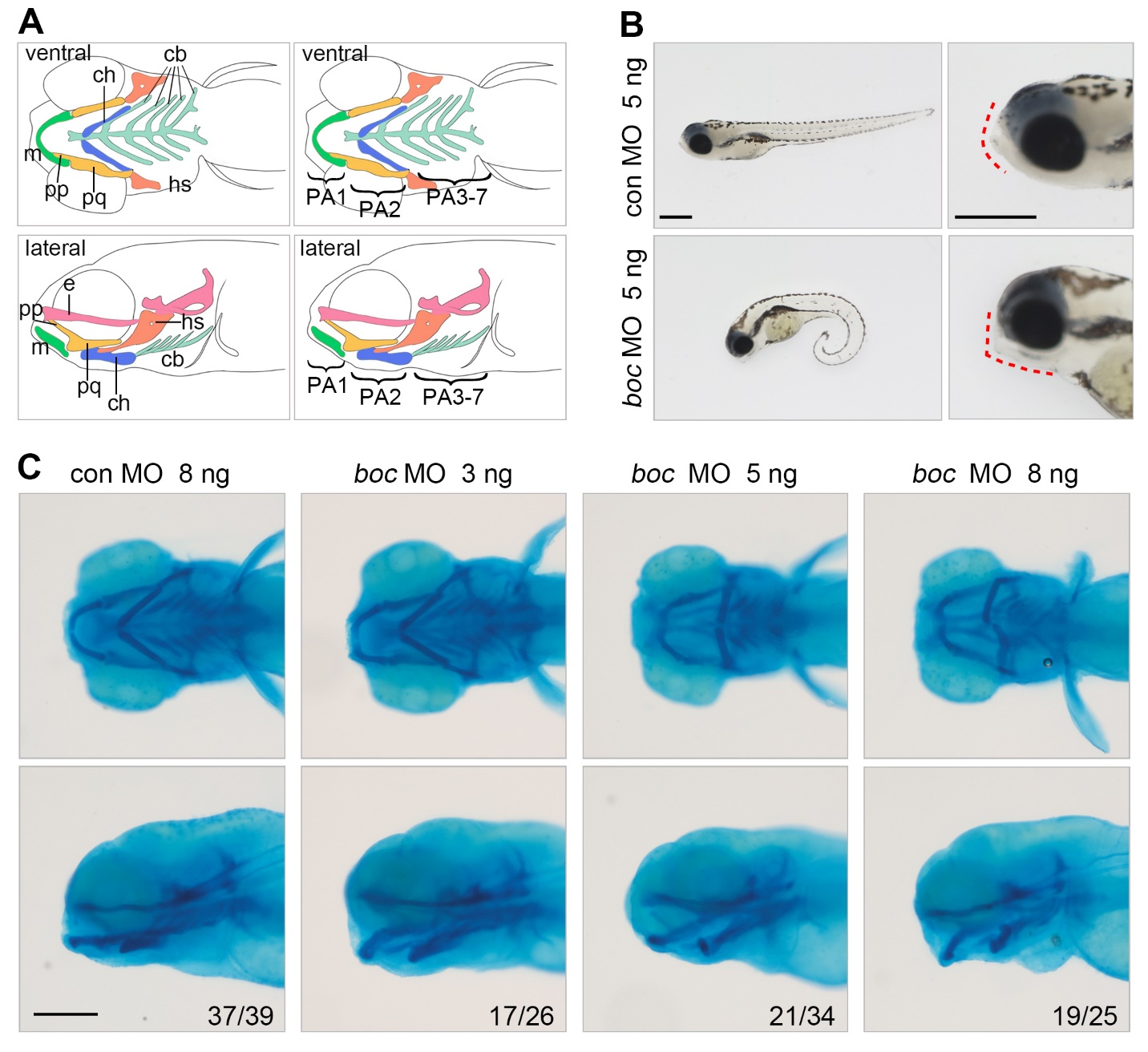
**

**Figure S7. Dose-dependent effect of *boc* MO on zebrafish craniofacial development.** (A) Schematic illustration of zebrafish craniofacial cartilage. m, Meckel’s cartilage, green; pp, pterygoid process of the palatoquadrate, orange; pq, palatoquadrate, orange; ch, ceratohyal, blue; cb, ceratobranchial; e, ethmoid plate; hs, hyosymplectic; PA, pharyngeal arch. (B) *boc* MO causes curved axis and jaw defects. 1-cell stage zebrafish embryos were injected with 5 ng of control MO or *boc* MO. Gross morphology of embryos was analyzed at 5 dpf. The head region in left panels was magnified and shown in the right panels. Scale bars: 200 μm. (C) Representative craniofacial cartilage defects caused by *boc* MO. 1-cell stage zebrafish embryos were injected with increasing dosages of control MO or *boc* MO and analyzed by Alcian blue staining at 5 dpf. 3 ng of *boc* MO injection resulted in 65.4% of zebrafish larvae exhibiting abnormal deviation of Meckel's cartilage growth direction towards the ventral side. Upon 5ng or 8ng of *boc* MO injection, Meckel’s cartilage exhibited a widened overall morphology with abnormal growth direction skewed towards the ventral side. This concurrently led to severe malformation in the development of palatoquadrate, ceratohyal, and ceratobranchial structures, resulting in significant craniofacial developmental abnormalities. The numbers of affected embryos and total embryos were indicated. Scale bars: 200 μm.


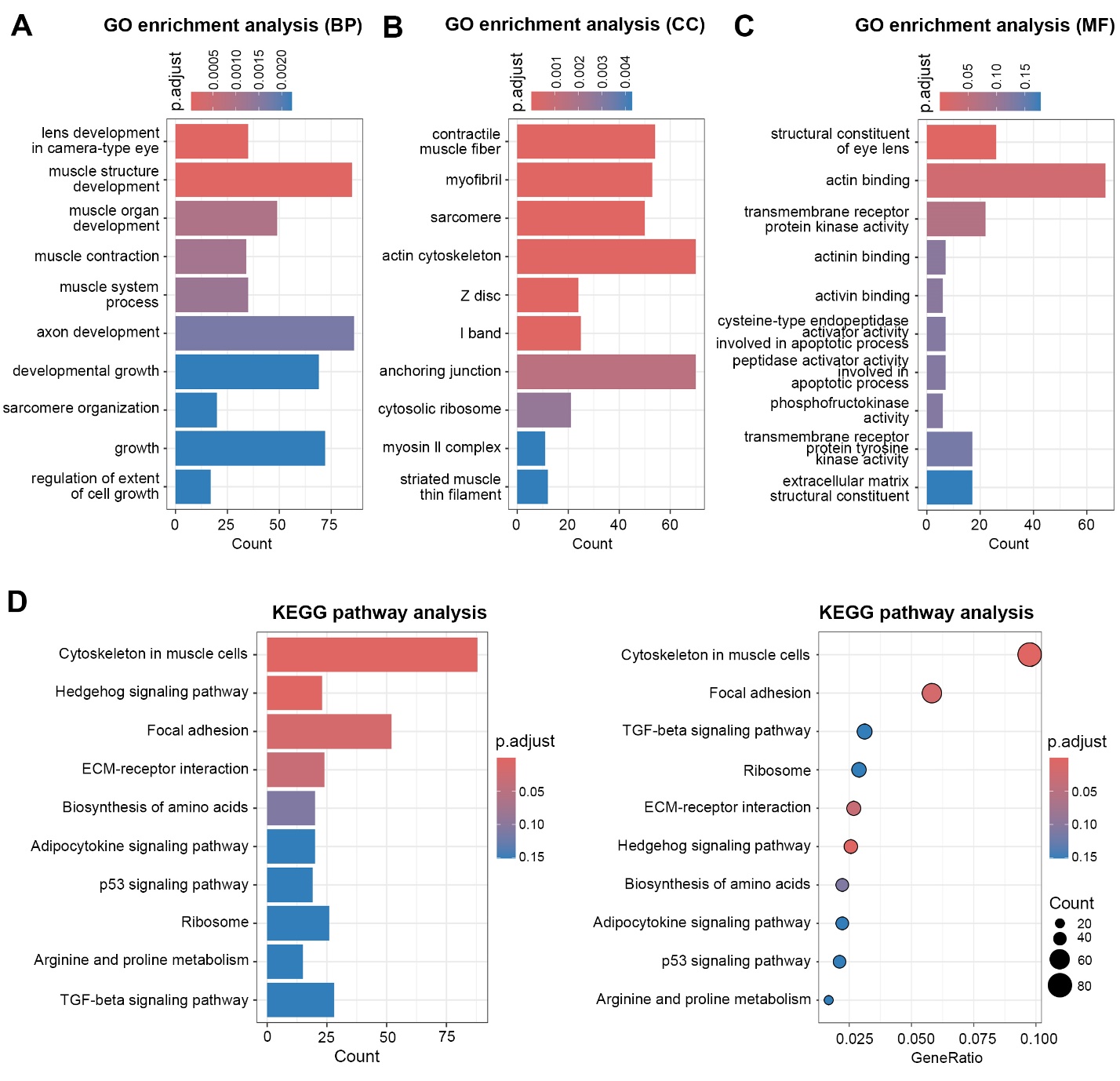


**Figure S8. Functional enrichment analysis of differentially expressed genes (DEGs) from RNA-seq data.** (A-C) Gene Ontology (GO) enrichment analysis showing significantly altered terms in (A) biological processes, (B) cellular components, and (C) molecular functions. (D) KEGG pathway analysis of dysregulated signaling pathways. Column color intensity represents the statistical significance (-log_10_[P-value]), with warmer colors indicating greater significance. Top 10 most significant terms are shown for each category (FDR < 0.05).

**
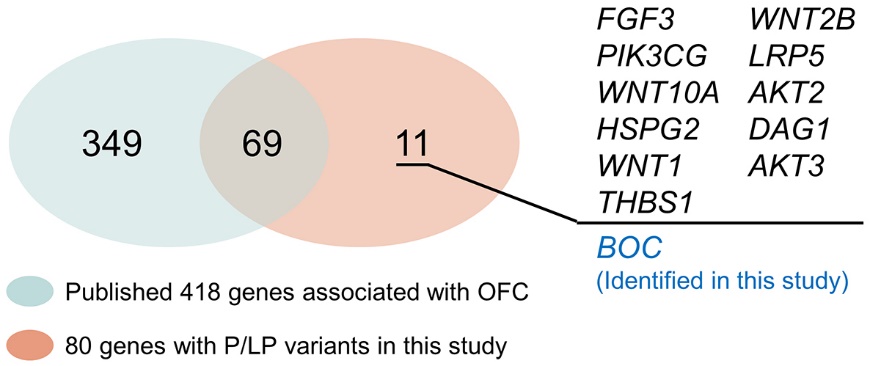
**

**Figure S9. An updated version of OFC gene list.** Aside from the published 418 OFC genes, 12 more genes were added to the list, including: *FGF3*, *PIK3CG*, *WNT10A*, *HSPG2*, *WNT1*, *THBS1*, *WNT2B*, *LRP5*, *AKT2*, *DAG1*, *AKT3* and *BOC* (identified in this study).
