# Supplementary material for: Exome Sequencing Reveals the Genetic Architecture of Non‐syndromic Orofacial Clefts and Identifies BOC as a Novel Causal Gene
Source: Adv Sci (Weinh). 2025 Jun 4;12(32):e12073. doi: 10.1002/advs.202412073 (PMC12407381; doi:10.1002/advs.202412073)
Supplement: Supplementary file 2 — Supporting Information [file ADVS-12-e12073-s002.docx]

| Population | Gender | Phenotype | Count | Ratio (%) |
| --- | --- | --- | --- | --- |
| Han Chinese | Male | CL | 38 | 17.76 |
|  |  | CP | 53 | 24.77 |
|  |  | CLP | 13 | 6.07 |
|  | Female | CL | 43 | 20.09 |
|  |  | CP | 58 | 27.10 |
|  |  | CLP | 9 | 4.21 |

**Supplemental Table 1. Demographic and phenotypic characteristics of 214 sporadic patients with NSOFCs.** NSOFC, non-syndromic orofacial cleft; CL, cleft lip; CP, cleft palate; CLP, cleft lip and palate
