# Supplementary material for: Exome Sequencing Reveals the Genetic Architecture of Non‐syndromic Orofacial Clefts and Identifies BOC as a Novel Causal Gene
Source: Adv Sci (Weinh). 2025 Jun 4;12(32):e12073. doi: 10.1002/advs.202412073 (PMC12407381; doi:10.1002/advs.202412073)
Supplement: Supplementary file 6 — Supporting Information [file ADVS-12-e12073-s003.docx]

**Primer sequences for BOC and GLI2 variant verification by Sanger sequencing**

| Primer name | Primer sequence (5’-3’) | Amplicon size | Variant |
| --- | --- | --- | --- |
| BOC-1219-F | GCCAAGCTTACCTGTGAGGTGC | 451bp | p.R407W |
| BOC-1219-R | GTGGTTGTCAAACTGGGTTCCACAG |  |  |
|  |  |  |  |
| BOC-1306-F | GTGGCGGTACAGCTGATGGTG | 422bp | p.G436S |
| BOC-1306-R | CCACATAGTAGAGGATTGGCGCC |  |  |
|  |  |  |  |
| BOC-3052-F | GGGAATGATGGTGTGGTACCTCTTG | 491bp | p.D1018N |
| BOC-3052-R | CACATAAATGCTGTGGCTGGCTCC |  |  |
|  |  |  |  |
| BOC-2041-F | GCGGGTGTTCTTGTGA | 293bp | p.R681X |
| BOC-2041-R | GCATCCGTGAAGGTGA |  |  |
|  |  |  |  |
| GLI2-1628-F | TGCGTGTCAGCAGTAGC | 656bp | p.A543G |
| GLI2-1628-R | GGAAATTGAGGCACAGAG |  |  |

**Primer sequences for qRT-PCR validation of RNA-seq data (targeting *bcl2a*, *mycn*, *ccnd1*, *ccnd2a*, *ccnd2b* and *jag2a*)**

| Primer name | Primer sequence (5’-3’) | Amplicon size |
| --- | --- | --- |
| *bcl2a*-F | GTTTGGTGGGACCATGTGCG | 237bp |
| *bcl2a*-R | GTCACTCCTGCCAAGCCCAG |  |
|  |  |  |
| *mycn*-F | GGAGCAGCAGCAGCCAAAG | 200bp |
| *mycn*-R | GGAACGCCTCTTCTCCACCG |  |
|  |  |  |
| *ccnd1*-F | GGAAAATCGTCGCGACGTGG | 142bp |
| *ccnd1*-R | GCTCCTAAAAGCTGCAACCTGG |  |
|  |  |  |
| *ccnd2a*-F | GTTTGCTGACCATCGAAGAGAGG | 222bp |
| *ccnd2a*-R | CGCCGAGCAACTGCAAGTTAC |  |
|  |  |  |
| *ccnd2b*-F  *ccnd2b*-R | CTTCTGCGATGAACGAGTATTGC | 257bp |
|  | GACAAACAGCTCCTAAGAGCTGC |  |
|  |  |  |
| *jag2a*-F | GAATGTCAGTCGTCTCCCTGTTC | 207bp |
| *jag2a*-R | GTTTCCATTCATGCACTGACAGC |  |
|  |  |  |

**Primer sequences for *bcl2a*/*mycn*/*ccnd1* promoter amplification in ChIP-qPCR**

| Primer name | Primer sequence (5’-3’) | Amplicon size |
| --- | --- | --- |
| *bcl2a*-F | GATGTTCATTAAAGTCGCTGGTG | 102bp |
| *bcl2a*-R | CTAACTGAATGTACAAGACATGG |  |
|  |  |  |
| *mycn*-F | GGTTTCACTCACTCACTATCCTG | 97bp |
| *mycn*-R | GAATGCCATCCGCTGCATTAC |  |
|  |  |  |
| *ccnd1*-F | GATGCTCAATTGGTACTAATGGG | 123bp |
| *ccnd1*-R | CCTCAACAACATGAGAGCATG |  |
